# Complete Genome Sequence of *Sporisorium scitamineum* and Biotrophic Interaction Transcriptome with Sugarcane

**DOI:** 10.1371/journal.pone.0129318

**Published:** 2015-06-12

**Authors:** Lucas M. Taniguti, Patricia D. C. Schaker, Juliana Benevenuto, Leila P. Peters, Giselle Carvalho, Alessandra Palhares, Maria C. Quecine, Filipe R. S. Nunes, Maria C. P. Kmit, Alvan Wai, Georg Hausner, Karen S. Aitken, Paul J. Berkman, James A. Fraser, Paula M. Moolhuijzen, Luiz L. Coutinho, Silvana Creste, Maria L. C. Vieira, João P. Kitajima, Claudia B. Monteiro-Vitorello

**Affiliations:** 1 College of Agriculture “Luiz de Queiroz”, University of São Paulo, Av. Pádua Dias 11, PO BOX 9, 13400-970, Piracicaba, São Paulo, Brazil; 2 Department of Microbiology, University of Manitoba, Winnipeg, Manitoba, Canada, R3T 2N2; 3 CSIRO Agriculture, Flagship, Queensland Bioscience Precinct, 306 Carmody Road, St Lucia, QLD, 4067, Australia; 4 Australian Infectious Diseases Research Centre, School of Chemistry and Molecular Biosciences, The University of Queensland, Brisbane, QLD, Australia; 5 Centre for Comparative Genomics, Murdoch University, Perth, Australia; 6 Centro Avançado da Pesquisa Tecnológica do Agronegócio de Cana—IAC/Apta Ribeirão Preto, São Paulo, Brazil; 7 Mendelics Análise Genômica, Rua Cubatão 86, Cj. 1602, 04013-000, São Paulo, SP, Brazil; Illinois Institute of Technology, UNITED STATES

## Abstract

*Sporisorium scitamineum* is a biotrophic fungus responsible for the sugarcane smut, a worldwide spread disease. This study provides the complete sequence of individual chromosomes of *S. scitamineum* from telomere to telomere achieved by a combination of PacBio long reads and Illumina short reads sequence data, as well as a draft sequence of a second fungal strain. Comparative analysis to previous available sequences of another strain detected few polymorphisms among the three genomes. The novel complete sequence described herein allowed us to identify and annotate extended subtelomeric regions, repetitive elements and the mitochondrial DNA sequence. The genome comprises 19,979,571 bases, 6,677 genes encoding proteins, 111 tRNAs and 3 assembled copies of rDNA, out of our estimated number of copies as 130. Chromosomal reorganizations were detected when comparing to sequences of *S. reilianum*, the closest smut relative, potentially influenced by repeats of transposable elements. Repetitive elements may have also directed the linkage of the two mating-type *loci*. The fungal transcriptome profiling from *in vitro* and from interaction with sugarcane at two time points (early infection and whip emergence) revealed that 13.5% of the genes were differentially expressed *in planta* and particular to each developmental stage. Among them are plant cell wall degrading enzymes, proteases, lipases, chitin modification and lignin degradation enzymes, sugar transporters and transcriptional factors. The fungus also modulates transcription of genes related to surviving against reactive oxygen species and other toxic metabolites produced by the plant. Previously described effectors in smut/plant interactions were detected but some new candidates are proposed. Ten genomic islands harboring some of the candidate genes unique to *S. scitamineum* were expressed only *in planta*. RNAseq data was also used to reassure gene predictions.

## Introduction

**Fig 1:**
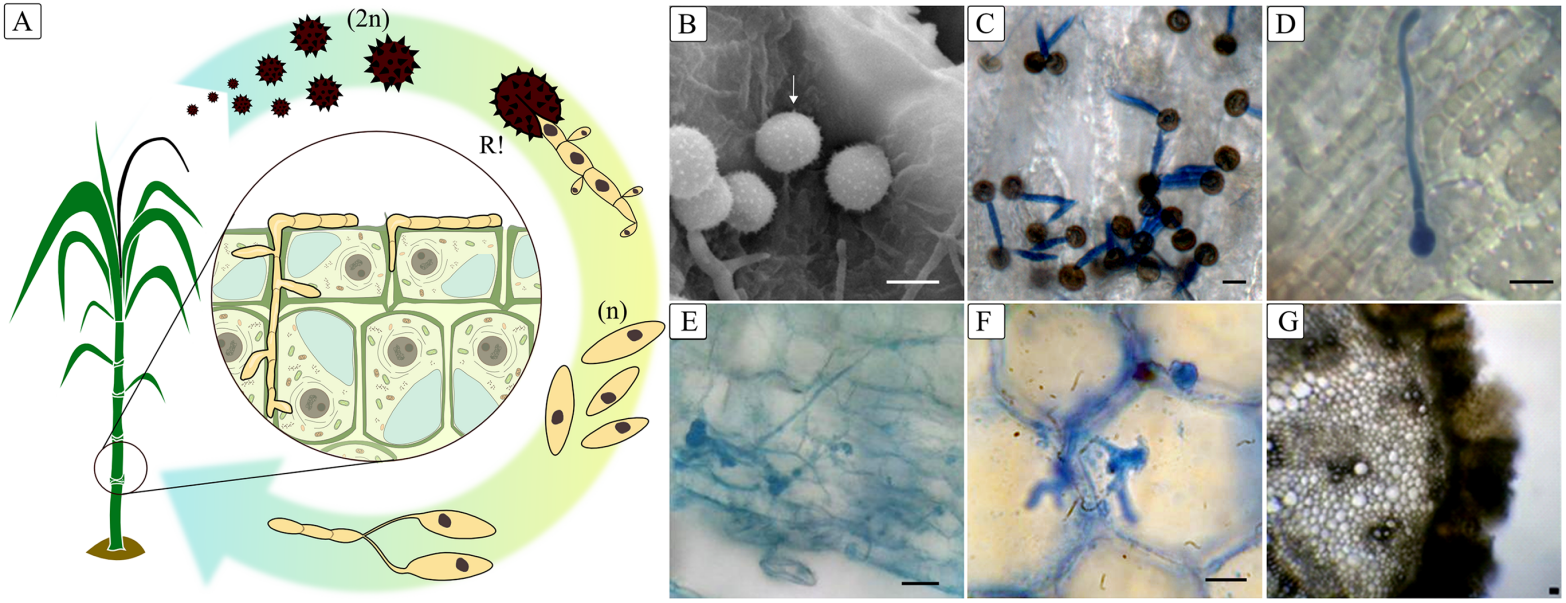
(A) Developmental stages in the *S. scitamineum* life cycle: diploid teliospores (2n); haploid yeast-like sporidia (n) after meiosis (R!); hyphal fusion. (B) Scanning electron micrograph of spores adhered to sugarcane bud surface. (C) Germination of spores on bud scale epidermis and tube-like promycelium formation at 6 hai (hours after inoculation); photomicrograph of tube-like promycelium stained with lactophenol-cotton. (D) Photomicrograph of apressorium formation 48 hai stained with lactophenol-cotton blue; (E) Photomicrograph of *S. scitamineum* growth on parenchyma cells of bud tissue observed at 120 hr stained with lactophenol-cotton blue. (F) Photomicrograph of *S. scitamineum* intracellular growth on parenchyma cells of white whip portion; stained with lactophenol-cotton blue. (G) Photomicrograph of black whip portion showing the mature spore liberation. Scale bar = 5 *μ*m

Sugarcane is an important crop worldwide due to its capability to store large amounts of sucrose in the stem, supplying more than half of the world’s sugar consumption for centuries [1]. Currently, it is also considered the most efficient bioenergy crop for ethanol production [2]. One important factor impairing the increase of sugarcane production is the severity of some diseases affecting the crop [3]. *Sporisorium scitamineum*, causal agent of the sugarcane smut disease, is a constant concern among producers and breeders, since the disease has been found at low levels in all cultivated areas. There are three distinct phases during the fungal life cycle: haploid yeast-like sporidia, dikaryotic hyphae and diploid teliospores (Fig 1). To infect the host, a combination of two haploid sporidia belonging to opposite mating-types is necessary to form an infective hyphae. Fungal hyphae differentiate appressorium structures to penetrate plant tissues. Sugarcane infected plants show a profound metabolic modification resulting in the development of a whip-like structure from the stalk apex composed of a mixture of plant and fungal tissues. Within these structures millions of dark teliospores develop and are responsible for disseminating the disease [4]. Under appropriate environmental conditions, teliospores germinate promycelia leading to meiosis and production of haploid sporidia that will become infective after the fusion of compatible sporidial cells [5]. Hyphal fusion and sexual cycle are crucial for disease establishment and dissemination. *In vitro*, *S. scitamineum* is maintained easily as colonies of yeast-like cells derived of budding sporidia and hyphal colonies, which facilitates genetic studies [6]. The only part of the life cycle restricted to host tissues so far is the development of teliospores. Sugarcane plants are prone to pathogen attack in the early stages of growth, however, whips are observed only after 45 days in more susceptible varieties. Infected sugarcane plants have abnormal grass-like growth, reduced culm diameter, and very fibrous stalks which are poor in sugar content [4].

Although little information is available from *S. scitamineum* biology, more is known about its close relative *Ustilago maydis*, a smut fungus of corn. Noteworthy in the latter, are a number of genes arranged in groups encoding uncharacterized secreted proteins associated with the control of fungal colonization in host plants [7]. Although among various smut fungi the genomes are similar in size and gene content, disease symptoms related to the formation of fungal sporangial structures and host specificity are unique to each smut species/plant interaction, and therefore comparative analysis may help to uncover genes that trigger specific host responses [8]. This work presents the fully sequenced genome of *S. scitamineum* SSC39B and a draft of isolates BSES, which enabled us to perform comparative analysis to another genome sequence recently published [9]. The complete sequence of each chromosome of *S. scitamineum* SSC39B also enabled comparisons regarding genome organization to the closest smut species *S. reilianum*[10]. Fungal transcriptome profiles during *in vitro* growth and throughout its life cycle *in planta* at two time points revealed candidate genes unique to the interaction with sugarcane.

## Materials and Methods

### Ethics Statement

*S. scitamineum* SSC39B haploid cells derived from a single mating-type were used for complete genome sequencing. The cells were obtained from a teliospore (SSC39) isolated from a whip developed in the sugarcane susceptible clone IACSP98-2053. The whip was collected from an experimental area of “Centro de Cana—Instituto Agronômico de Campinas” (21°12′04″S; 47°52′20″W), and no specific permissions were required for sampling diseased plants in this location. The isolates BSESs were isolated from a teliospore isolated from a whip developed in a susceptible sugarcane clone Q117. The whip was collected from an experimental field at the Sugar Research Australia pathology farm at Woodford, Australia (26°55′43”S; 152°46′38.3”E), no specific permissions were required for sampling diseased plants in this location.

### Strains and sequencing strategies

*S. scitamineum* haploid cells derived from a single mating-type were used for complete genome sequencing. A single yeast-like colony was isolated and mating-type was assigned as described in Bölker *et al*. (1992) [11]. The *S. scitamineum* SSC39B genome was sequenced using the long reads PacBio technology [12]. DNA was extracted using “DNeasy Blood and Tissue Kit” (Qiagen). Five SMRT cells (P5-C3 chemistry) were used in the sequencing process, generating 2.2 Gbp of raw data corresponding to more than 100 fold of sampling depth (738,568 reads with average length of 3,047 bp). Besides PacBio sequencing, paired-end libraries of the same strain were sequenced using Illumina HiSeq 2000. A whole cell DNA library was prepared for sequencing using the kit Nextera DNA Sample Preparation Workflow (Illumina paired-end). A total of 85,465,786 reads of 100 bp in length (approximately 8.5 Gb) corresponding to more than 400 fold of sampling depth were analyzed using FASTQC (ver. 0.10.1) and Seqyclean (ver. 1.8.10) to filter low quality sequences (phred quality below 20).

In addition, an isolate of the opposite mating-type (SSC39A) was partially sequenced using a BAC genomic library that was constructed using the protocol described by Peterson *et al*. (2000) [13] and the “CopyControlT BAC Cloning Kit” (Epicentre). The genomic library is composed of 2,880 clones with an insert size average of 92 kbp representing 10 X coverage of the genome. Forty-eight BAC clones were selected from pools of 30 plates (96 clones each). Included in this analysis were PCR amplifications for the mating-type genes using the enzyme “GenomiPhi” (GE Healthcare Life Sciences) in combination with following primers for: *Locus b* (UsibEF 5’ -GCTGGTCCAACATTCTCC- 3’ and UsibER 5’ -CGCTTGCTCTCTGCTTAG- 3’) and *Locus a* (SSC39AF 5’ -AGATCGGGAAGAAAATG-AGC- 3’ and SSC39AR 5’ -TTGTATCATCGTGGGTCTCTGG- 3’). Sequencing data were generated with Illumina HiSeq technology, also with paired-end reads of 100 bp in length. These reads were used to assemble the mating loci sequence of SSC39A.

Two additional Australian isolates, BSES15 and BSES17, which represent contrasting mating-types, were also sequenced using the Illumina platform. Illumina sequencing generated paired-end 90bp reads on an Illumina GAIIx and 5,588,889 read pairs (1.01 Gbp) and 4,335,980 read pairs (0.78 Gbp) were generated for isolates BSES15 and BSES17 respectively.

### Genome Assembly

Given the high coverage of PacBio reads, the HGAP.2 assembler [14] was used with default parameters. The resulting assembly was composed of 59 contigs (19 Mbp) and 11 singletons (88 kbp). A final genome version was built manually with the alignment results, generating 26 final contigs (genome V0). CLC Genomics Workbench V7.01 (CLC Bio) was then used to align all Illumina reads against this assembly. The consensus of the aligned Illumina paired-end reads (parameters: global alignment, minimum of 0.9 read length with minimum 90% identity) against the V0 genome was then saved as the V1 *S. scitamineum* genome. This assembly was aligned against the *S. reilianum* genome using cross_match (www.phrap.org). The mitochondrial genome was assembled individually (CLC Genomics Workbench V7.01) by using the mitochondrial sequence of *S. reilianum* as reference. The DNA data of BSES15 and BSES17 was analyzed using the Blue error correcting algorithm [15] and assembled using the Velvet Optimiser algorithm of the Velvet assembler [16] at an optimal k-mer length of 77.

### Pulse field gel electrophoresis and hybridization

DNA plugs for pulse-field gel electrophoresis were prepared using CHEF Genomic DNA Plug Kit (Bio-Rad Laboratories Inc.) as described previously [17]. Fragments from 200 to 2200 kbp were separated in contour-clamped homogeneous electric field electrophoresis, conducted in a CHEF II (Bio-Rad Laboratories Inc.) apparatus. Pulse-field electrophoresis was carried out as follows: a 1% agarose gel in 0.5x TBE buffer was held at 14°C by a temperature-controlled cooling unit for 15 h, at 6 V x cm^-1^, electric current was regulated with the initial pulse = final pulse = 70 s, and a second step for 11 h, 6 V x cm^-1^ (initial pulse = final pulse = 120 s). Size of bands were estimated by the software Kodak Digital Science 1D 3.0.2, using Pulse Marker 225-2200 kbp (Sigma-Aldrich Corp.). Hybridizations were performed using “AlkPhos” Direct Labeling and Detection System’ (GE). Probes used were sequences of a telomere insert of pTEL13 [18] and a PCR amplicon containing the rDNA internal transcribed spacer region (ITS1, 5.8S and ITS2) of *S. scitamineum* generated with primers: Hs 5’ -AACACGGTTGCATCGGTTGGGTC- 3’ and Ha 5’ -GCTTCTTGCTCATCCTCACCACCAA- 3’) [19].

### Genome and Proteome Annotations

Gene prediction was accomplished by using the *U. maydis* gene model calculated by the Augustus software [20] integrating the RNAseq paired-end reads (see further below). The tRNAs were identified by the tRNAscan-SE program [21]. Repetitive elements were identified using RECON [22], setting BLASTn to a cutoff of e-value ≤ 1 × 10^-10^. Only families with more than 15 members with sequences ranging from 500 to 2,500 bp were considered to be repetitive elements. BLASTx searches against UNIREF50 were performed to identify transposable elements containing one of the following PFAM domains: PF00078, PF01498, PF03108, PF03184, PF03221, PF10551, PF13359 [23]. RepeatMasker [24] annotation supplied with our custom library (RECON + RepBase v. 19.11) was used to define repetitive elements.

Telomeric repeats were identified by the Tandem Repeat Database (TRDB) tool [25]. The length and localization of each telomere as well as the subtelomeric predicted genes within the chromosome ends (50 kbp) were manually checked.

Predicted proteins were analyzed using the Blast2Go tool V2.7.2 (BLASTp with cutoff e-value < 1 x 10^-5^) [26] and Fisher’s exact test was used to detect enrichment of GO terms (p-value ≤ 0.05). The programs SignalP V4.0.1c [27], TMHMM V2.0c [28] and predGPI [29] were used to predict those proteins that are potentially secreted and to define the secretome. The HMMs from dbCAN [30] were used to predict and classify the CAZymes. Reference proteins of the PHI-base (Pathogen-Host Interaction database) [31] were used to obtain experimental evidences to our predicted proteins using BLASTp e-value < 1 x 10^-14^ and query coverage of more than 80%.

The mitochondrial genome was annotated with the MFannot program combined with RNAweasel [32]. The potential *rnl* gene and putative ORFs, including intron/exon junctions, were further resolved with ORFfinder and by comparative sequence analysis using the BLAST suite of programs and by aligning intron containing genes with orthologs that lack insertions. Alignments were performed with the MAFFT program [33] with settings that allow for long insertions and short conserved regions and these alignments were manually adjusted with the Genedoc program to narrow intron/exon junctions [34]. Intergenic regions were examined with ORFfinder to identify potential remnants of ORFs, in particular eroded homing endonuclease coding segments.

### Comparative genome analysis

Synteny studies between chromosomes of *S. reilianum* and *S. scitamineum* were performed using BLASTn (cutoff e-value ≤ 1 x 10^-5^) combined with Circos [35] to draw the relationships between chromosomes. In addition the complete genome of strain SSC39B was compared to the BSES assembly using cross_match. The scaffolds from the genome assembly of the strain 2014001 (Genbank: JFOL00000000.1) [9] was aligned using MUMmer (V3.0), and BEDTools [36] merging local similarities in a range of 1,500 bp of the genome alignment.

Comparisons of predicted coding sequences among the three strains were performed using BLASTn. Genes particular to the SSC39B were defined by considering either “no hit” or an e-value > 1 x 10^-5^.

The *S. scitamineum* SSC39B proteome was compared to all predicted proteins of *U. maydis*, *U. hordei* and *S. reilianum* (MIPS database [37]). OrthoMCL with default parameters was used to determine the orthologous groups among the proteomes [38]. Visualization of the OrthoMCL results was obtained with a four-way-Venn-diagram drawn in R language using the Venn diagram package [39].

Phylogenetic analysis of mating-type protein sequences were performed based on a multiple sequence alignment generated by T-COFFEE [40]. The best amino acids substitution model that fits the data was determined by using the Akaike Information Criterion (AIC) in the software ProtTest v3.2 [41]. Maximum likelihood trees were obtained for each protein considering the heuristic method NNI (Nearest Neighbor Interchange) for searching through treespace and aLRT SH-like (approximate Likelihood-Ratio Test with Shimodaira-Hasegawa-like procedure) for quantifying branch support using PhyML v3.0 [42]. *S. scitamineum* protein sequences of both mating-types and sequences for close related species available on GenBank were included in the analysis.

### Transcriptome assay and analysis

The *S. scitamineum* SSC39 teliospores (> 90% viability) were mixed with saline solution and used to inoculate sugarcane plants of the smut susceptible variety “RB92-5345”. Single budded sets of 7 month-old plants were surface disinfected, heat treated (52°C for 30 min in water bath, 1 kg of buds/6L of water) and incubated for 16 h at 28°C. Sets were then placed on trays with buds facing upwards, and inoculated using the wound-paste method [43]. Pots were kept in the greenhouse arranged into a completely randomized experimental design. Fungal transcriptome profiles were obtained 5 days after inoculation (DAI) from tissues of the breaking buds and at 200 DAI from the base of the whip-like structure emission (where intense fungal cell division and sporogenesis occurs). For *in vitro* transcriptome analysis, haploid yeast-like cells of opposite mating-types were grown separately in liquid medium [44] in a orbital shaker for 15 h at 28°C. Cells of both mating-types were mixed prior to RNA extraction. All samples were frozen in liquid nitrogen immediately after collection and stored at -80°C. Three biological replicates were systematically used.

Total RNA extraction from 5 DAI samples was performed using the lithium chloride based method [45, 46]. TRIzol Reagent (Life Technologies, UK) was used for RNA extraction from 200 DAI samples and control cells. DNA was extracted from the same 5 DAI samples to confirm infection before the construction of RNAseq libraries. The rDNA ITS region was amplified with primers Hs and Ha [19] to confirm the presence of *S. scitamineum*.

Libraries were constructed following Illumina manufacturer’s protocol of the “TruSeq RNA Sample Prep v2 Low Throughput (LT)” kit. Paired-end sequencing was performed on the Illumina platform (HiScanSQ). Reads were analyzed by FASTQC (ver. 0.10.1) and low quality bases (phred ≥ 20), Illumina adapters and poly-A tails were removed using the Seqyclean program (ver. 1.8.10). The RNAseq fungal reads from the 5 DAI and 200 DAI plant materials were recovered from the total reads by mapping to the complete genome of *S. scitamineum* SSC39B strain using Bowtie2 [47]. RNAseq reads were also aligned to all *S. scitamineum* coding sequences, using Bowtie2 with default parameters to determine the % of CDSs length coverage.

Differential transcript accumulation among treatments (5 DAI and 200 DAI and controls) was observed using the CLC Genomics Workbench V7.01. Fungal reads were mapped to CDSs of *S. scitamineum* (100% of nucleotide identity and 98% of coverage). The mapping of at least one read pair in all three replicates was considered to be a positive match. Scaling approach as implemented in the CLC software was used as the normalization method. Baggerley’s test and the false discovery rate (FDR) with a significance level of ≤ 0.01 and Log_2_FoldChange ≥ 2 or ≤ -2 (treatment/control) were applied to generate a set of differentially expressed genes. Transcripts were considered specific to the interaction with sugarcane if at least one pair of reads mapped to all three replicates of each of the treatments and none to the control. Enrichment test of GO terms were performed with the BLAST2GO tool using the two-side Fisher’s Exact Test (p-value < 0.05).

## Results

### General Results

PacBio sequencing allowed us to determine the complete sequence of *S. scitamineum* SSC39B chromosomes. The final assembly comprises 19,979,571 nucleotides distributed in 26 contigs (no gaps within contigs). Telomere motifs were identified for 23 chromosomes longer than 475 kbp in length (Table A in S1 File). The other three contigs (24, 25, 26) have 87,293, 82,225 and 99,152 kbp, respectively, and are also found in the assemblies of 2014001 and BSESs genomes (Figure A in S1 File). The results of PFGE hybridized with telomeric probes (Figure B in S1 File) confirmed the presence of a DNA band of around 100 kbp. Sequence analysis revealed that these contigs are composed of repetitive elements (Figure A in S1 File) and have few predicted genes supported by RNAseq. At this time remains to be established if all of the three assembled contigs are chromosomes of *S. scitamineum* or if they are misassembles of one or more small chromosomes.

The rDNA cluster was found to be close to the end of chromosome numbered 23 and three copies were assembled. Hybridization data (Figure B in S1 File) together with the coverage of Illumina reads allowed us to estimate that there were 130 copies of rDNA units comprising the rDNA region. Unexpectedly, the length of the chromosome harboring the rDNA sequence varied between the two isolates of the same genetic background but with opposite mating types (Figure B in S1 File). This result was confirmed using hybridizations with probes prepared from two independent regions of the rDNA (data not shown). The rDNA gene cluster is composed of 5,979 bp (18S—5.8 S—28S) and the first copy is located at coordinates 119,074 of chromosome 23. The 5 S rDNA genes are scattered among the chromosomes and 14 copies were identified. Similarly, 111 tRNA genes corresponding to all 20 amino acids were found to be dispersed among the chromosomes (Table B in S1 File).

**Fig 2:**
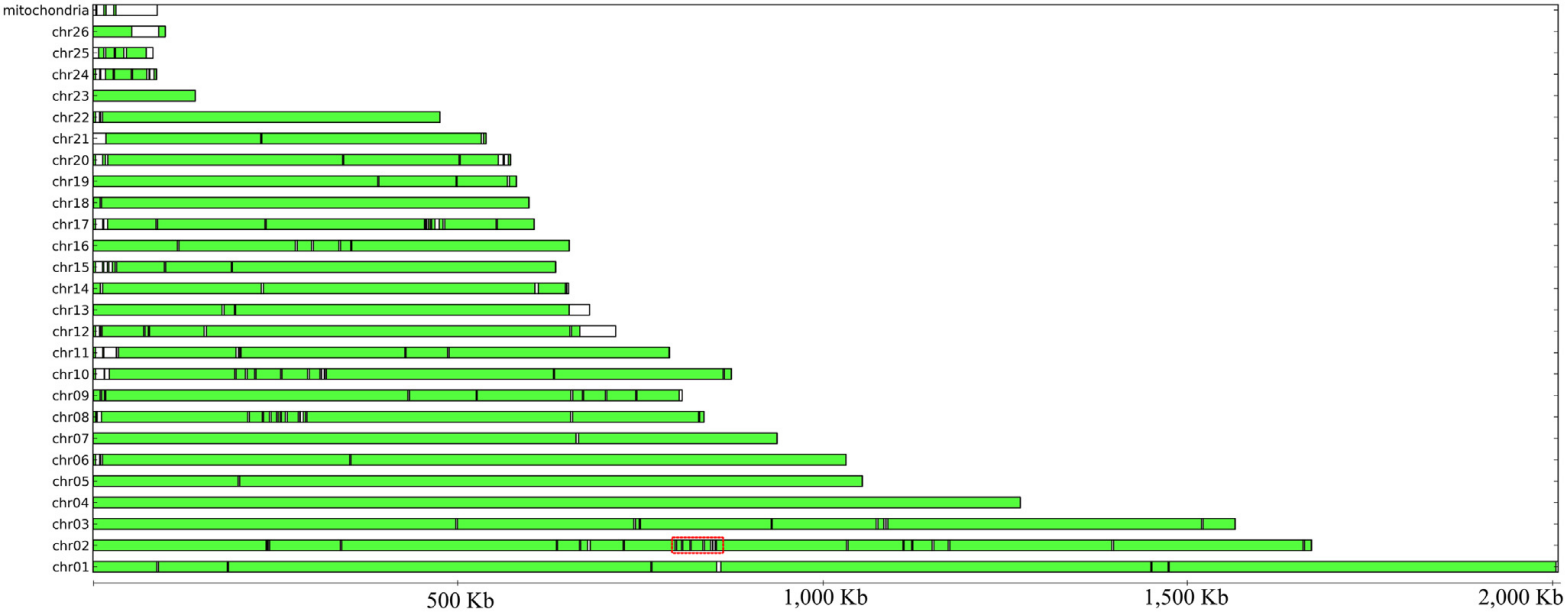
Representation of all chromosomes and contigs assembled of the strain SSC39 genome compared to the strain 2014001 scaffolds. Regions present in the strain 2014001 are shown in green blocks, delimited by black borders. White regions represent sequences unique to the strain SSC39 assembly. The analysis was performed using MUMmer 3 and parameters = -maxmatch -c -L -b -l 500. Red line above the chromosome 2 indicates the region containing the mating-type *loci*

Assemblies of the BSES15 and BSES17 isolates generated 5,331 contigs. A total of 19,234,547 bp (99%) aligned to the strain SSC39B genome with the following variation: 0.075% substitutions, 0.007% deletions and 0.038% insertions. The comparison between the genome sequences of the SSC39B and 2014001 strains (the last presenting 19,619,026 bp organized in 35 scaffolds [9]) showed that most of the chromosomes assembled are covered. However, the mitochondrial genome was poorly represented in the 2014001 assembly and nuclear genome derived scaffolds do not extend over repetitive regions as well as subtelomeric regions (Fig 2).

**Fig 3:**
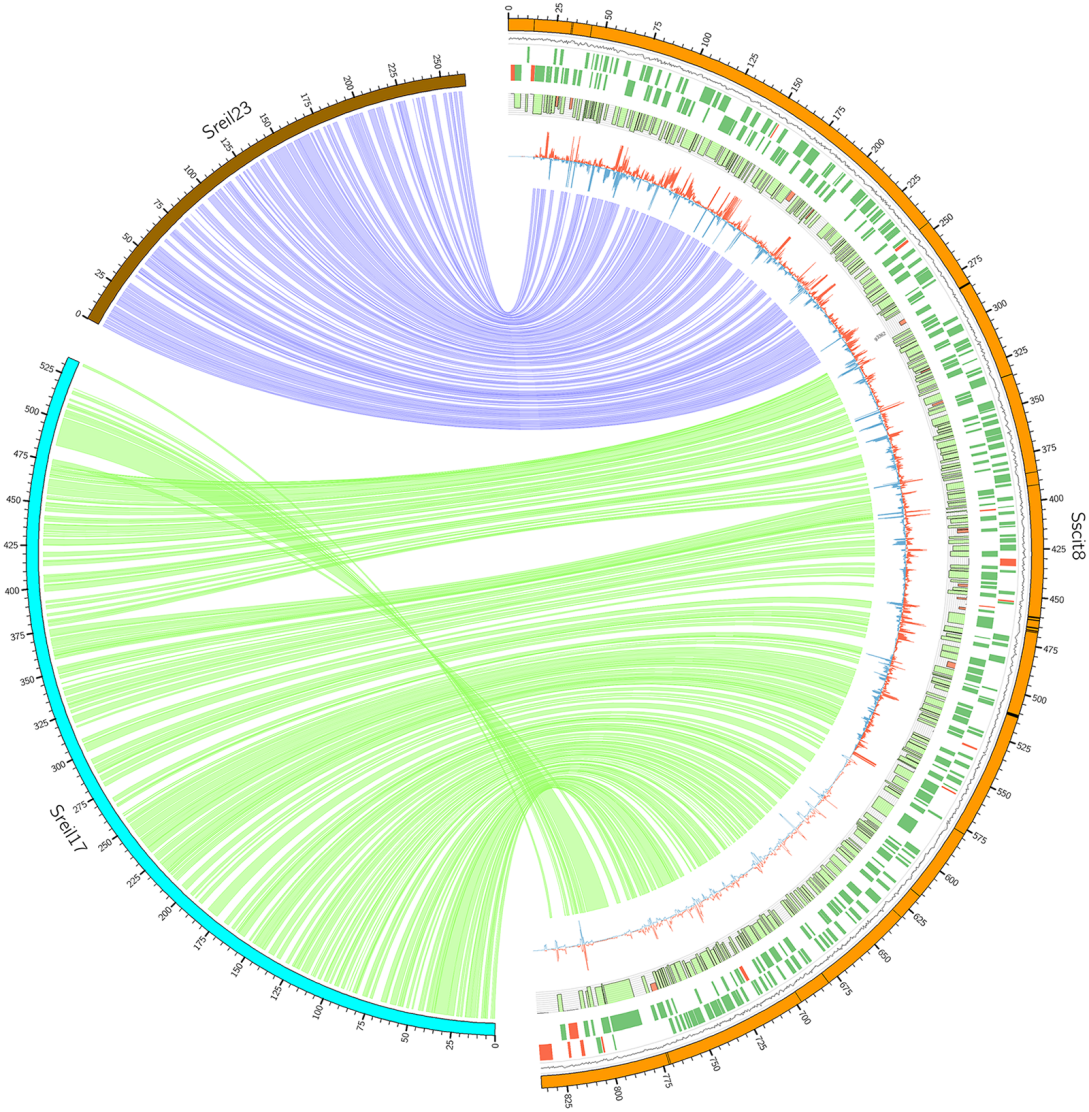
Syntenic view of two chromosomes of *S. reilianum* that merged as one in *S. scitamineum*. Links represents alignment length of more than 1 kbp obtained by BLASTn (e-value < 1 x 10^-5^). The first outer circle represents the chromosome and scale is coordinates in base pairs. The second indicates the GC content followed by predicted coding regions of the plus and minus strands. Bars display the % of identity to orthologous in *S. reilianum*. The most inner circle represents the RNAseq coverage of each chromosome region. Red lines are RNAseq data of *S. scitamineum* growing in planta and blue lines growing *in vitro*. Circle images of all chromosomes are available in the Supporting Information S3 File.

The *S. scitamineum* strain SSC39 genome comprises 6,677 protein coding genes, with an average number of introns per gene of 1.94, and 51% of the genes (3,415) have no introns (Table C in S1 File). Most of the CDSs are between 500 and 2,000 kbp in length (3,861) and only a few are larger than 7 kbp (34) (Figure C in S1 File). In general most of the CDSs have an ortholog in the *S. reilianum* genome (Fig 3).

Of the total *S. scitamineum* predicted genes, 6,479 (97%) are supported by mapping the reads produced by RNAseq analysis. A total of 198 have no coverage, but 72 of these have significant matches (BLASTn e-value ≤ 1 x 10^-14^ and query coverage ≥ 75%) to putative CDSs of *U. maydis*, *S. reilianum* or *U. hordei* genomes. Repetitive elements represented 1.24% of the *S. scitamineum* genome. Gene Ontology was used to assign GO terms to the set of predicted proteins to improve the organization of the annotation data [26]. GO terms were successfully assigned to 3,682 proteins (55.2%) (Table A in S2 File). As expected, top hit species results (5,078) are with *S. reilianum* proteins (Figure B in S2 File).

### Telomeric and subtelomeric regions

Telomeric arrays of tandem repeats were localized at both ends of all chromosomes, with an average repeat length of 76 bp that range from 32 bp to 140 bp (Table A in S1 File). The subtelomeric gene set comprises 800 predicted genes coding for proteins. Thirty-five helicase genes located in 22 chromosome ends were found, which include 3 predicted telomere-linked RecQ-helicase (TLH) genes, a typical feature of subtelomeric regions. As observed in other fungi, a notable group of genes related to niche adaptation (carbon utilization and catabolism) and pathogenicity were also reported (Table D in S1 File). Yet, some of the uncharacterized genes located near the chromosome ends are potentially associated with interaction with sugarcane (discussed later) (Fig 3).

### Chromosomal rearrangements and mating-type analysis

**Fig 4:**
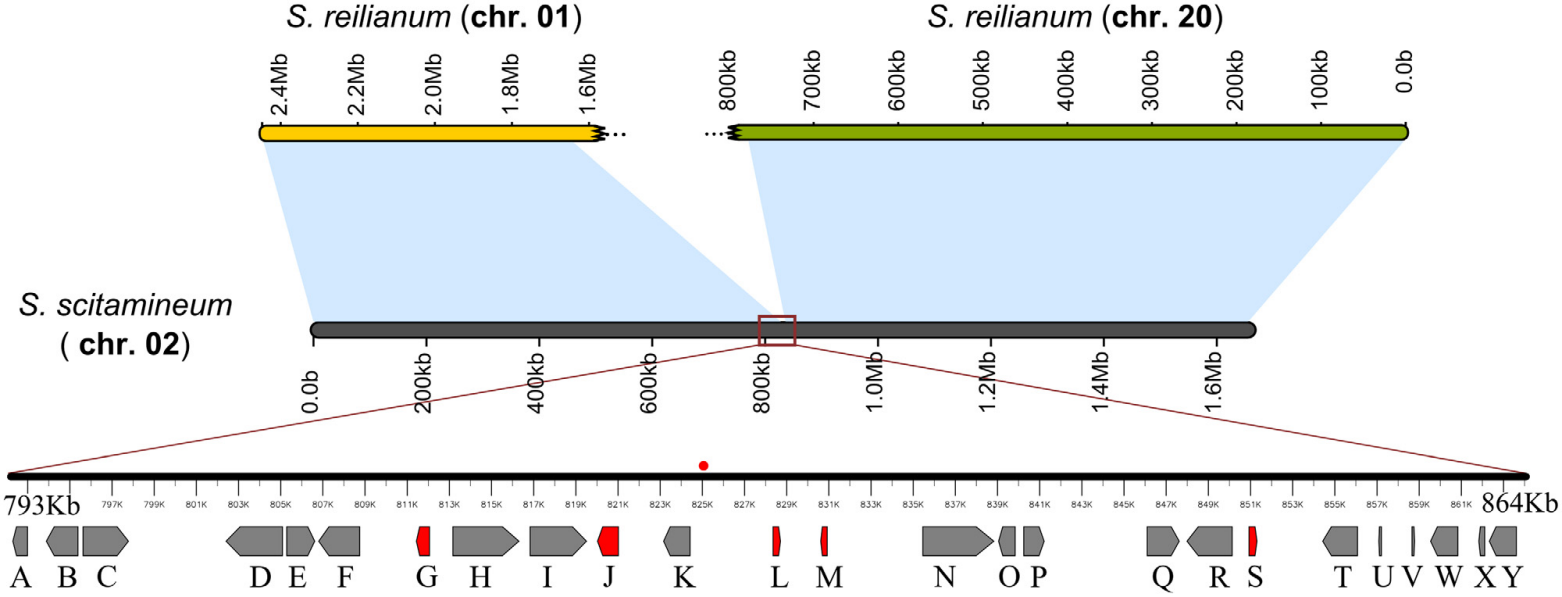
Blocks of synteny between chromosome 2 of *S. scitamineum* and chromosomes 1 and 20 of *S. reilianum* and schematic representation of the linked mating-type *loci* in *S. scitamineum*. Blue areas correspond to syntenic regions considering BLASTn e-value ≤ 1 x 10^-5^. Red lines represent the expansion of the region containing the mating type genes in *S. scitamineum* located at positions 792,295 bp to 863,606 bp of the chromosome 2. The chromosome breakpoint is identified and indicated by a red dot above the sequence. Genes are indicated by gray arrows placed according to transcriptional orientation and the transposons related sequences are highlighted in red. Letters represent functional annotation of encoded proteins: A) *c1d1* putative nuclear regulator; B and C) homeodomain transcription factor *bE1* and *bW1*, respectively; D) *nat1* putative N-terminal acetyltransferase; E, F, M, N, P, Q and R) Uncharacterized protein; G, J, M and S) Related to transposase; H) *sla*—cytoskeleton assembly control protein; I) RPN5-26S proteasome regulatory protein; K) *hhp1* casein kinase-1; L) related to reverse transcriptase; O) *arp2/3*—actin related protein 2/3 complex; T) *lba1* left border *a* *locus*; U) and V) pheromone gene *mfa1.2* and *mfa1.3*, respectively; W) *pra1* pheromone receptor gene; X) *Rba2*—right border *a locus*; Y) *pan1*—pantoate-beta-alanine ligase.

Comparative genomics showed that among the four smut fungi genomes used here *S. scitamineum* and *S. reilianum* are the closest, with an average nucleotide identity of 85.4% among all predicted CDSs. Eight *S. scitamineum* chromosomes display different arrangements with regards to *S. reilianum* as well as breaking points enriched with transposable elements (Figs. in S3 File). One of such breaking points is a region relevant to the biology of *S. scitamineum* that links the two mating-type *loci* referred to as *a* and *b*. In *S. scitamineum*, both *loci* are located 59 kbp apart on chromosome 2, characteristic of a bipolar mating system [48] (Fig 4). Chromosome 2 of *S. scitamineum* is homologous to chromosomes 1 and 20 of *S. reilianum*, which also harbor the mating-type *loci* *b* and *a*, respectively, in a tetrapolar arrangement [49]. Pairwise comparisons of these chromosomes revealed that the breakpoint occurred at the position 825,021 of *S. scitamineum* chromosome 2 (Fig 4). Within this inter-mating-type *loci* several remnants of DDE_1 and LINE transposons were noted. In addition, 20% of the sequence in between bases 801,345 and 854,308 is composed of repetitive elements, a much larger percentage when compared to the respective 1.24% of the genome.

The mating-type *loci* of the SSC39B complete genome sequence are equivalent to MAT1 of *U. hordei* and a1 of *U. maydis*, as determined by the similarity to genes of the mating *loci* (Figure A in S4 File). Based on the percentage of sequence identity, phylogenetic analysis and gene organization comparisons (Figures A and B and Table A in S4 File), the mating-type proteins of close and distantly related smut species differ substantially. BLASTp analysis shows that predicted proteins from whole genome of *S. scitamineum* have, on average, 82.5, 75.4 and 72.4% of identity with proteins of *S. reilianum*, *U. maydis* and *U. hordei*, respectively. In contrast, their mating-type proteins have lower percentages of identity (Table A in S4 File).

Phylogenetic analysis was performed including other smut mating-type protein sequences extracted from databases and proteins encoded by the opposite mate pair of *S. scitamineum* obtained by sequencing BAC genomic library inserts (Figure B in S4 File). Proteins encoded by *b locus* (*bE1/bE2* and *bW1/bW2*) of *S. scitamineum* (SSC39A and SSC39B) are more close related to each other than to the *bE* and *bW* proteins from related species; whereas proteins encoded by its *a locus* (*pra1/pra2* and *mfa1.2/mfa1.3/mfa2.1/mfa2.3*) cluster preferentially with alleles from others species (Figure A in S4 File). The genomic context indicates that the order and orientation of the genes in the *b locus* are conserved (Figure B in S4 File). Otherwise for the *a locus*, these settings vary among species and between alleles of the same species, especially by the presence of *rga*2 and *lga*2 genes in the *a*2-type alleles that are absent in the *a*1-type (Figure B in S4 File). The *a locus* of *S. scitamineum* and *S. reilianum* also differs from that of *U. maydis* and *U. hordei* by the presence of a second pheromone gene, named mfa1.3 in *S. scitamineum* due to its similarity to the pheromone mfa2.3 from *S. reilianum*. It is noteworthy that this extra copy of mfa in *S. scitamineum* lacks the characteristic CAAX motif (C, cysteine; A, aliphatic amino acid; X, any amino acid) at its carboxyl terminus. Pheromone response elements (PREs), with a binding motif “ACAAAGGGA” for transcription factor prf1, were found close to the mating-type genes in *S. scitamineum* and in the others fungi analyzed, with the exception of *Malassezia* species (Figure B in S4 File).

### The mtDNA Annotation

The mitochondrial genome has 88,018 bp and presents the standard 14 protein coding genes (*nad2, nad3, nad4, nad4L, nad5, nad6, cox1, cox2, cox3, cob, atp2, atp6, atp9* and *rps3*) along with 11 unknown hypothetical ORFs (Table A and B in S5 File). The last nucleotide of the *nad2* gene is the first one of the *nad3* gene. Among the RNA encoding genes, 22 tRNA genes were detected representing all 20 amino acids, and two tRNAs for each Ser and Met; the *rns* and *rnl* genes were also present and finally two potential copies of the *rnpB* gene were noted. However one copy is interrupted by tRNA(V) and may represent an eroded copy of the gene or a misannotation based on the presence of analogous RNA folds that resemble components of the *rnpB* encoded RNA molecule. Among the various genes 24 group I introns were noted, of these 23 encoded putative ORFs. The *cox1* gene was the most intron rich gene with 11 insertions. Nineteen LAGLIDADG type endonuclease ORFs were noted and among these ORFs four showed evidence of degeneration, i.e. the fragmentation due the presence of premature stop codons. Four intron ORFs belong to the GIY-YIG family of homing endonucleases and two appear to have eroded, as above due to the accumulation of mutations and the presence of premature stop codons. A few eroded freestanding GIY-YIG ORFs were noted in the intergenic regions but these were quite fragmented and thus not annotated. The *atp9*, *cox1*, *nad1*, *nad5*, and *nad6* genes were all followed by duplicated versions that lacked the N-terminal components. Differences between the complete version of the gene and the partial duplicated version fragments of GIY-YIG type ORFs could be detected by programs such as ORFfinder or by BLASTx.

### Comparative proteome

A total of 6,475 groups of homologs were described among the four smut species analysed (*S. scitamineum, S. reilianum, U. maydis, U. hordei*), each one containing at least two proteins (Table A and Figure A in S6 File). The largest intersection was the one comprising the four species, with 5,507 orthologous groups (85.05%), among them, 5,347 containing exactly one ortholog from each proteome. *S. scitamineum* proteome contains few groups harboring paralogs, thus, most of their genes are single copy (94.3%) (Table 1). The largest paralog family has 61 members related to ATP-dependent DNA helicase (RecQ), some of which identified in close vicinity of telomeres as mentioned previously.

**Table 1:** Comparative analysis of orthology among four smut fungi obtained by OrthoMCL.

**Genome characteristics**	***S. scitamineum***	***S. reilianum***	***U. maydis***	***U. hordei***
Protein-coding genes	6,677	6,675	6,784	7,111
Co-orthologs groups	6,061	6,271	6,135	5,971
Genes into the groups	6,328	6,443	6,378	6,649
In-paralogs genes	267	172	243	678
Singletons	349	232	406	462
Single-copy genes	6,298 (94.3%)	6,365 (95.4%)	6,372 (93.9%)	6,257 (88%)

The OrthoMCL analysis resulted in a few unique orthologous clusters for each species that are represented by potentially duplicated genes (Table B in S6 File) and in a higher number of singletons (genes not assigned to any OrthoMCL cluster), which represent single copy genes unique to each species (Table 1). Most of the *S. scitamineum* unique genes (23 clusters including 89 proteins and 349 singletons) are related to transposable elements and uncharacterized proteins, but RNAseq reads suggest that some of these genes are active at the transcriptional level (Table C in S1 File). Among genes with predicted functions, there are for example, putative effectors related to the Mig1 gene family (g3918_chr10_Ss and g3919_chr10_Ss) and a group of six paralogs related to sterol delta 5,6-desaturase (g2628_chr06_Ss, g3812_chr10_Ss, g4371_chr12_Ss, g6420_chr22_Ss, g6606_chr24_Ss and g6623_chr24_Ss) (Table B in S6 File).

### Secretome

The *S. scitamineum* genome encodes 527 predicted proteins showing a positive match to signal peptides, of these 342 have no transmembrane domains and 305 are also not anchored by GPI. These proteins were selected to compose the secretome of *S. scitamineum*. We used the HMMs from dbCAN to identify 54 CAZymes in the secretome, including eight containing carbohydrate binding modules (CBM), 11 carbohydrate esterases (CE), 30 glycoside hydrolases (GH), one glycosyl transferase (GT), one polysaccharide lyase (PL) and six with auxiliary activities (AA) (Table F in S1 File). Nearly 30% of secretome (93 proteins) have at least one GO term assigned according to Blast2GO results (Figure A in S7 File).

The secretome of *S. scitamineum* is composed of 148 (48.5%) predicted proteins of uncharacterized function. Moreover, 38 of these proteins are considered unique to *S. scitamineum* by OrthoMCL analysis (Table B in S6 File), wherein 29 proteins are singletons and nine proteins belong to three orthologous clusters.

**Fig 5:**
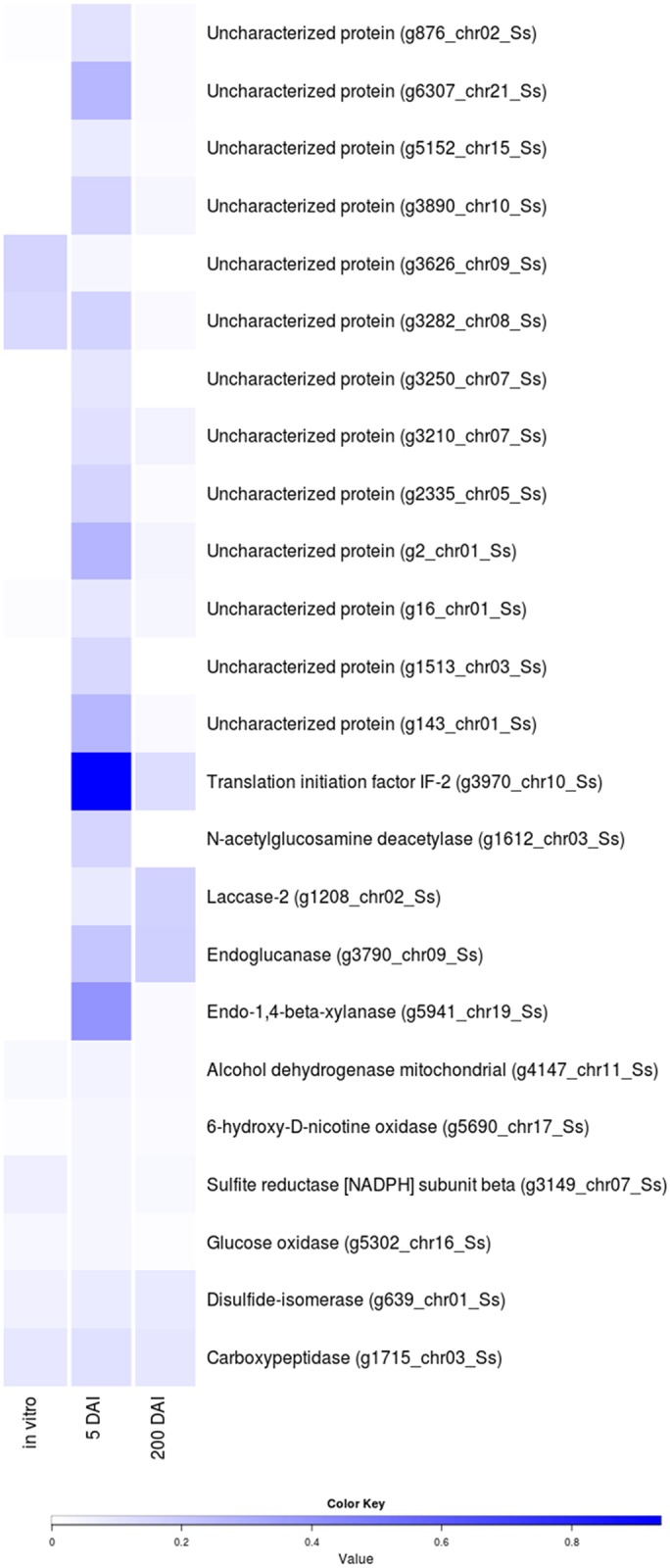
Genes encoding secreted proteins and their expression level under all three conditions: *in vitro*, 5 DAI, 200 DAI as calculated by CLC Genomics Workbench. Heatmaps were obtained using the function heatmap.2 of the package gplots in R language

We analyzed genes of the secretome expressed at 5 DAI and have constructed a heatmap showing the modulation of these genes under the three tested conditions (Fig 5). According to the functional annotation results, they encode hydrolases, peptidases, oxidases and reductases as well as several genes of unknown functions. The modulation of gene expression is particular to each time point during the interaction with sugarcane and to *in vitro* growth.

### Transcriptome Analysis

The data revealed that 56.6%, 41.3% and 54.7% of RNAseq trimmed reads mapped in pairs to *S. scitamineum* CDSs *in vitro*, 5 DAI and 200 DAI, respectively (Table A in S8 File). The number of CDSs detected in each experiment was 6,213 (93%) *in vitro*, 437 (6.5%) at 5 DAI and 6,183 (92.6%) at 200 DAI. The RNAseq data were analyzed based on a combination of expression change threshold of Log_2_FoldChange (treatment/control) ≥ 2 or ≤ -2 among treatments, and relied on FDR as defined by CLC software. In addition, considering the relatively low number of fungal reads recovered from the assay *in planta* at 5 DAI, we have analyzed the most expressed genes in a given treatment considering the number of mapping reads per CDS normalized by scaling and CDS length. In this case, only genes mapped by at least one pair of reads in all three replicates were taken into account. We called these genes preferentially expressed (Table B in S8 File). The mapping levels of reads to genes preferentially expressed were between 6 and 10 times higher than the average number of reads mapped per CDS.

The number of preferentially expressed genes encoding secreted proteins is higher *in planta* (16) than *in vitro* (3). Among them, only two genes were in common to the two time points analyzed *in planta* (5 DAI and 200 DAI): the endoglucanase (g3790_chr09_Ss) and an uncharacterized secreted protein (g3890_chr10_Ss). Most of the genes considered preferentially expressed *in vitro* encode proteins related to energetic metabolism and growth, including an alternative oxidase (g2905_chr06_Ss), which is the most expressed, ATP-ADP carrier protein, elongation factor 1-alfa, polyubiquitin and several ribosomal proteins.

The results of the differentially expressed genes, obtained comparing each treatment *in planta* to the *in vitro* control, resulted in 125 genes detected at 5 DAI, of these 119 are up-regulated and six down-regulated *in planta* (Table C in S8 File). At 200 DAI 907 genes were detected as differentially expressed, of these 641 are up-regulated and 266 down-regulated (Table D in S8 File). GO terms assigned to down-regulated genes at 5 DAI are enriched in members of the mRNA binding functional group, and processes related to carbohydrate metabolism, oxidation-reduction and cellular respiration (Table E in S8 File). Up-regulated genes at 5 DAI are enriched in functions related to transporter activity and molecular/signal transduction (Table C in S8 File). At 200 DAI, the most enriched GO terms among down-regulated genes are oxidoreductase activity and mitochondrion cellular component. Up-regulated genes at 200 DAI are enriched with terms of hydrolase activity acting on glycosyl bonds and carbohydrate metabolic process (Table D in S8 File).

Among differentially expressed genes up-regulated *in planta* (5 and/or 200 DAI) 78 encode secreted proteins, some of them are related to host attack, nutrient acquisition and chitin modification. Table 2 summarizes the most relevant proteins detected as differentially expressed.

**Table 2:** List of selected differentially expressed genes up-regulated in planta. For complete list view Tables C and D in S8 File.

**Genes encoding secreted proteins**			
	**Gene ID**	**Gene product**	**Time point**
Host attack	g74_chr01_Ss	Related to pepsin (Aspartate protease)	200 DAI
	g189_chr01_Ss	Related to Lipase	200 DAI
	g252_chr01_Ss	Glucan 1,3-beta-glucosidase	200 DAI
	g468_chr01_Ss	Probable beta-glucosidase	200 DAI
	g1208_chr02_Ss	Laccase-2	200 DAI/5 DAI
	g1656_chr03_Ss	Alpha-L-arabinofuranosidase	200 DAI
	g1624_chr03_Ss	Guanyl-specific ribonuclease	200 DAI
	g2264_chr04_Ss	Alpha-L-arabinofuranosidase	200 DAI
	g2858_chr06_Ss	Probable lysozyme	200 DAI
	g3042_chr07_Ss	Related to subtilisin-like serine protease	200 DAI
	g3262_chr08_Ss	Related to aminopeptidase	200 DAI
	g3529_chr08_Ss	Related to Pectin lyase	200 DAI
	g3568_chr09_Ss	Related to secreted aspartic protease	200 DAI
	g3696_chr09_Ss	Endo-1,6-beta-D-glucanase	200 DAI
	g3790_chr09_Ss	Endoglucanase	200 DAI/5 DAI
	g3919_chr10_Ss	Related to Mig1 protein	200 DAI
	g4618_chr13_Ss	Lipase	200 DAI
	g5316_chr16_Ss	Probable beta-glucosidase	200 DAI
	g4719_chr13_Ss	Probable pectinesterase	200 DAI
	g5941_chr19_Ss	Endo-1,4-beta-xylanase	200 DAI
	g6000_chr19_Ss	Glucan 1,3-beta-glucosidase	200 DAI
Nutrient acquisition	g4081_chr10_Ss	Related to 3-phytase	200 DAI
	g5690_chr17_Ss	6-hydroxy-D-nicotine oxidase	5 DAI
Chitin modification	g1612_chr03_Ss	Probable Chitin deacetylase	5 DAI
	g1900_chr04_Ss	Chitinase	200 DAI
	g6059_chr20_Ss	Related to Chitin-binding protein	200 DAI
Detoxification	g6307_chr21_Ss	Chorismate mutase	200 DAI
**Genes encoding not secreted proteins**			
siderophore transporters	g3806_chr09_Ss	Siderophore iron transporter	200 DAI
ammonium and nitrate transporters	g4863_chr14_Ss	Nitrate transporter	200 DAI
	g1183_chr02_Ss	High affinity ammonium transporter	5 DAI
	g6016_chr19_Ss	Glutathione transporter	200 DAI
	g5527_chr17_Ss	Ammonium transporter	5 DAI
amino acids and vitamins transport	g5482_chr16_Ss	Dityrosine transporter	200 DAI
	g2895_chr06_Ss	Probable metal-nicotianamine transporter	5 DAI
	g5681_chr17_Ss	Riboflavin transporter	200 DAI
sugar transporters	g4185_chr11_Ss	Hexose transporter	200 DAI/5 DAI
	g1478_chr03_Ss	Sugar transporter	200 DAI/5 DAI
	g1034_chr02_Ss	High-affinity glucose transporter	200 DAI
	g4185_chr11_Ss	Hexose transporter	200 DAI/5 DAI
	g1478_chr03_Ss	Sugar transporter	200 DAI/5 DAI
	g6532_chr22_Ss	UDP-galactose transporter	200 DAI
Invertase	g1777_chr03_Ss	Invertase	200 DAI
Detoxification	g4103_chr11_Ss	Salicylate hydroxylase	200 DAI
	g4198_chr11_Ss	Pisatin demethylase	200 DAI
Toxin biosynthesis	g3941_chr10_Ss	Versicolorin B synthase	200 DAI
Signal transduction	g2874_chr06_Ss	Hybrid signal transduction histidine kinase	5 DAI
	g1321_chr03_Ss	Serine/threonine-protein kinase	5 DAI
	g2134_chr04_Ss	Serine/threonine-protein kinase	5 DAI
	g2002_chr04_Ss	Probable serine/threonine-protein kinase	200 DAI
	g3652_chr09_Ss	Transcription initiation factor IIA large subunit	5 DAI
	g1400_chr03_Ss	Transcriptional activator of proteases	200 DAI
	g3766_chr09_Ss	Transcription factor RFX4	200 DAI
	g722_chr02_Ss	Serine/threonine-protein kinase	200 DAI
	g1809_chr03_Ss	Transcriptional regulatory protein	200 DAI

Searching for fungal genes expressed only *in planta* we found one gene specific to the interaction at 5 DAI (g4078_chr10_Ss), which encodes an uncharacterized protein, not secreted and with no conserved domains detected. Expressed only at 200 DAI are 131 genes: six of them (g5153_chr15_Ss, g5152_chr15_Ss, g5155_chr15_Ss, g3771_chr09_Ss, g4550_chr12_Ss, g3890_chr10_Ss) are also expressed at 5 DAI (Table F in S8 File), 108 encode proteins of unknown function, and 38 encode proteins of the secretome. The GO terms enrichment analysis revealed that extracellular region is the prevalent term. This set of 132 fungal genes is probably related to fungus/host interaction and may contain effectors associated with this singular interaction. Yet, the presence of one gene expressed particularly at 200 DAI encoding a secreted cysteine-protease inhibitor (g2337_chr05_Ss) may be related to the fungal defense against plant proteases. Eight of these genes have homologues in the PHI-base (Table G in S1 File), strengthening its involvement in *S. scitamineum* pathogenicity. For instance, mutations in *U. maydis* orthologs of genes g5161_chr15_Ss (PHI:932), g2659_chr06_Ss (PHI:910) and g3271_chr08_Ss (PHI:23) led to reduced virulence, and in the ortholog of g672_chr01_Ss (PHI:907) led to pathogenicity loss of the maize pathogen [7, 50].

**Fig 6:**
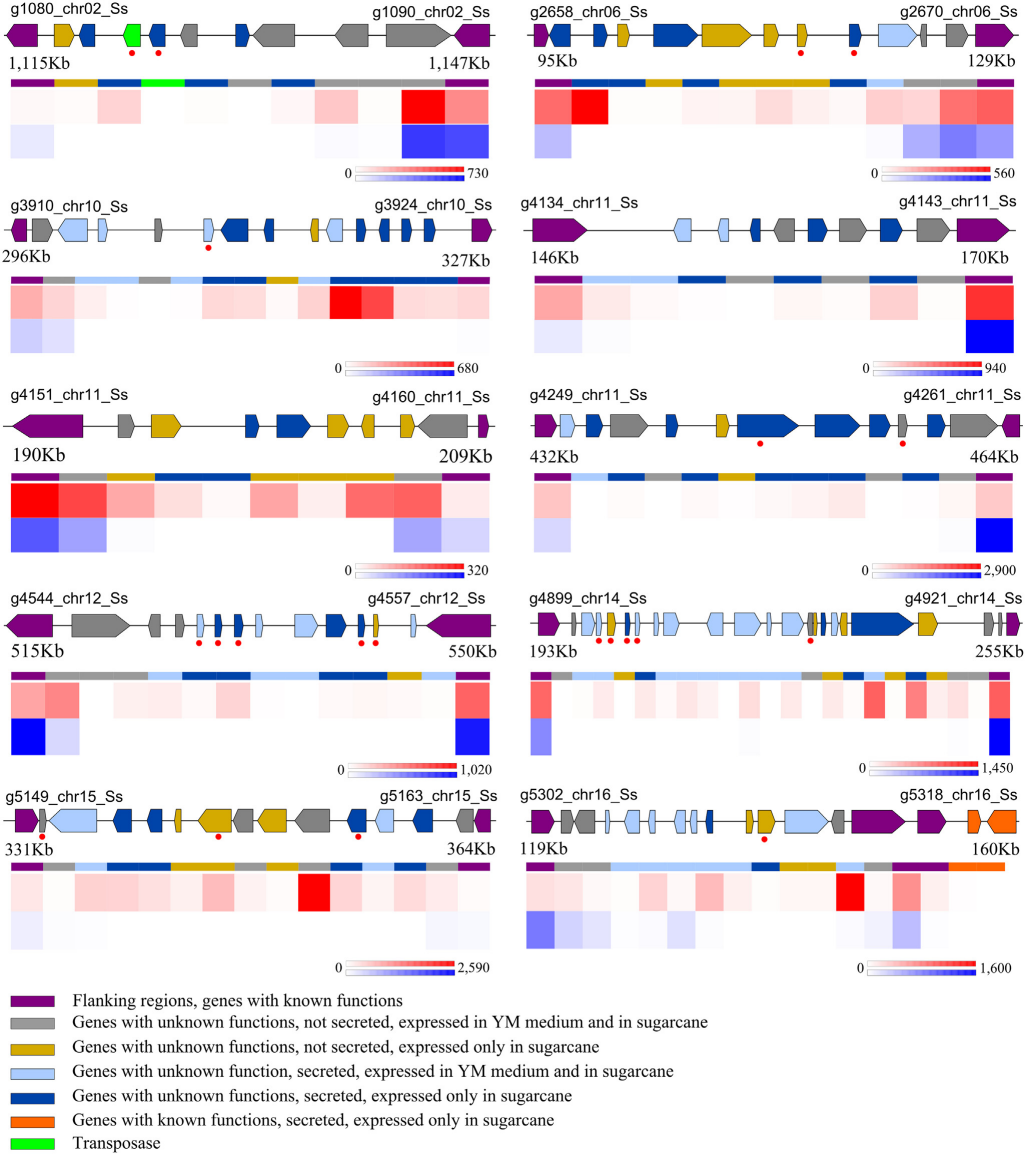
Chromosome segments representing the organization of genes in islands (color coded arrows and note colors beneath the bars). Expression at 200 DAI (heatmap red scale) and *in vitro*(heat map blue scale) are compared using the normalized number of mapped Illumina paired end reads, represented by the scales under each chromosome island. Gene names are presented at the borders of each segment of the chromosome, numbers represent the coordinates of these islands in kbp and red dots represent singlets as defined by OrthoMCL

The genome context of the genes expressed particularly *in planta* revealed the presence of ten islands in chromosomes 2, 6, 10, 11, 12, 14, 15 and 16 (Fig 6). Most of the genes are of uncharacterized function and encode secreted proteins ranging in size from 114 to 1257 amino acids. Mig1 related genes are in the chromosome 10 island and genes of the protein family Eff1 effectors are in an island of chromosome 11. Orthologous for 27 (22.9%) of these genes were not found in the genome of *S. reilianum*, the closest species, as well as in *U. maydis* and *U. hordei*, according to OrthoMCL analysis.

## Discussion

### The genome of *S. scitamineum*

The present study describes the successful use of deep coverage sequencing data combining long and short read strategies to achieve the complete sequence from telomere to telomere of each chromosome of the sugarcane smut pathogen *S. scitamineum*. Comparisons of the three available genomes of *S. scitamineum*, the SSC39 and BSES strains determined in this work and the published sequence of the strain 2014001 [9] showed few polymorphisms. However, we uncovered new information by revealing repetitive elements, subtelomeric regions and the mitochondrial DNA sequence. We also analyzed the chromosomal arrangement in comparison with the genome of the closest related species *S. reilianum*. Although, genomes of smut fungi are poor in repetitive elements compared to other fungi, the sequences associated with breaks in synteny between the two genomes revealed the presence of eroded transposable elements. We did not find complete copies of these elements. The genome of *S. scitamineum* is highly compact but similar to the genome sizes of other smut fungi [51] as well as to the gene predictions described for *U. maydis*, *S. reilianum* and *U. hordei* genomes that shows respective 6,675, 6,902 and 7,111 genes. Both the *S. reilianum* and *U. maydis* genomes were described as harboring 1% of transposable elements and *S scitamineum* harbors 1.24% in contrast to *U. hordei* that contains 3% of such elements [8].

We defined the location and number of rDNA repeat copies and surprisingly, based on hybridizations, there is a significant variation in chromosome length containing the rDNA cluster between the two mating types of the same genetic background. It has been reported that the copy number of the rDNA genes can vary spontaneously before meiosis [52]. The significance of this difference remains to be studied in *S. scitamineum*.

Besides the hallmark of telomeric encoded helicases, the subtelomeric sequences harbor genes potentially associated with host adaptation and pathogenicity, a common characteristic among fungi [53, 54]. Although this has not been shown for smut, some of the genes identified were related to sexual cycle, differentiation, sporulation, and to toxin detoxification and resistance. Subtelomeric regions are prone to contain polymorphic regions within genomes of different strains since telomere and subtelomere repeated sequences might favor rearrangements [55, 56]. Investigation of these regions among isolates may offer a better understanding of genetic diversity within fungal populations. Genes encoding proteins of unknown function arranged in clusters were also found within the edges of subtelomeric regions. Some of these regions are indeed associated with host/parasite interactions with sugarcane, since transcriptomic data showed the expression of these genes only *in planta*.

### mtDNA

The *S. scitamineum* mtDNA was determined to be comprised of 88,018 bp. The largest mitochondrial genome currently reported is from *Rhizoctonia solani* with 235,849 bp (NC_021436), which is in contrast to the relatively small mtDNA in the fission yeast *Schizosaccharomyces pombe*, with 19,431 bp (NC_001326). Among the Ustilagionomycotina reported mtDNAs sizes range from 29,999 bp (*Jaminaea angkoriensis*; KC628747.1) to 90,496 bp (*Sporosorium reilanum*; FQ311469.1). Very few mitochondrial genomes are available for members of the Ustiloginales (*U. maydis*, DQ157700.1; *Melanopsichium pennsylvanicum*, HG529787; and *S. reilianum*) and sizes range from 5,814 bp to 90,496 bp. Despite the size variation, the gene content among these genomes are very similar and size variability is mostly due to spacers and the presence or absence of introns and intron encoded ORFs [57–59].

The *S. scitamineum* mtDNA contains the 14 core protein coding genes found in all Basidiomycota mitochondrial genomes, encoding hydrophobic subunits of the electron transport chain, components of the ATPase synthase and the 40S ribosomal protein S3 [57]. RNA coding genes included the two ribosomal RNAs (*rnl* and *rns*), 22 tRNAs and the *rnpB* gene, which encodes the RNA component of the RNase P ribonucleoprotein involved in tRNA biogenesis.

Only group I introns were noted and these were located within the following genes: *rnl*, *nad5*, *cob*, *nad1*, *atp9* and the intron-rich *cox1* gene encompassing 11 ORF encoding introns. The 11 hypothetical genes (orphans that lack any counterparts in the NCBI database) identified by MFannot do not contain any introns. Mobile introns are characterized by their ability to “home” into cognate alleles that lack introns, therefore these elements tend to insert into conserved sequences which allows for more frequent lateral movement during crosses or during transient hyphal fusion among different species permiting cytoplasmic transfer [60]. The mobility is facilitated by the intron encoded proteins which are named homing endonucleases (HE) and in fungal mitochondrial genomes they can be assigned into two classes: LAGLIDADG and GIY YIG type HEases.

As stated earlier, several examples (*atp9*, *cox1*, *nad1*,*nad5* and *nad6*) were noted in which C-terminus of a gene is duplicated and located downstream of the full length version. These types of duplications were observed for some genes in the mtDNA of *Rhizoctonia solani*[57]. It might be significant that we have noted that GIY-YIG type ORFs are located between the full length version of the gene and the partial duplicated version. These arrangements could be the result of a GIY-YIG HEG invading the 3’ terminus of a gene thereby displacing the C-terminus coding part of the resident gene. But as it has been seen previously, when freestanding homing endonuclease genes (HEGs) invade genes they can also mobilize a segment that essentially compensates/duplicates the displaced segment [60–62]. As HEGs appear to be neutral elements and thus are not subject to selection, they quickly accumulate mutations [63] and thus only a few detectable fragments can now be recorded. In general based on these partial gene duplications associated with GIY-YIG types ORFs and other eroded GIY-YIG ORFs noted in the intergenic spacers it appears HEase activity contributes towards mtDNA size and organization.

### Mating-type analysis

Mating genes are crucial factors for disease establishment since a successful mating reaction is needed to form the infective dikaryotic hyphae. Both processes, mating and pathogenicity, are regulated by two *loci*: *a* and *b*[64]. The *a* *locus* comprises genes encoding a pheromone-receptor system necessary for cell-cell recognition and hyphal fusion, whereas the *b* *locus* comprises two genes encoding subunits of a heterodimeric homeodomain transcription factor regulating the maintenance of the dikaryon. Although considered to be phylogenetically close based on ITS and LSU rDNA analysis [65, 66], the two genera of smut fungi (*Ustilago/Sporisorium)* show high rates of amino acid substitution per site of the mating-type proteins. The trans-specific polymorphisms in pheromone and receptor genes are supposedly preserved since the last common ancestor of the basidiomycetes and ascomycetes and their reciprocal specificity likely have co-evolved [67]. Although the pheromone and receptor proteins differs among *Ustilaginaceae* species and seems to be optimized for intraspecific compatibility, interspecific sex up to the stage of plasmogamy can be still observed *in vitro*, which could have an evolutionary impact on speciation by hybridization events [67].

The genomic context of mating-type genes has been published for several smut fungi [9, 64, 67, 68]. The organization of the *b* *locus* genes is conserved among species in opposition to what has been observed for the *a* *locus*, even considering compatible mating-types with the same genetic background. *S. scitamineum* and *S. reilianum* differ from *U. maydis* and *U. hordei* by the presence of a second pheromone gene (*mfa)* in that *locus*. However, this extra copy (*mfa 1.3*) in *S. scitamineum* lacks the characteristic sequence acting as a signal for post-translational processing. The event involves the isoprenylation and carboxymethylation in the cysteine residue to form a secretable lipopeptide pheromone [69]. In *U. maydis*, a remnant of a pheromone gene is described for the *a2* allele that cannot produce a functional product [67, 70]. Although *mfa1.3* gene of *S. scitamineum* has full coverage of RNAseq reads, additional experiments are required to determine how many different mating-type alleles are in the fungal population and whether both pheromones are functional.

In *S. reilianum* and *U. maydis*, the mating-type *loci* are not linked and segregates independently (tetrapolar system) [68]; in *U. hordei* and *S. scitamineum* the *a* and *b* *loci* are linked and segregate as one *locus* (bipolar system) [9, 71]. Here we present the first completely sequenced intergenic region between the two mating *loci* among the smut genotypes and the occurrence of transposable elements at this region. In *U. hordei*, that also has a bipolar mating system, the scaffold that holds the mating-type *loci* has large stretches of long terminal repeats (LTRs) and transposable elements (TEs) dispersed within an intervening region of about 500 kbp [71]. This region may have been responsible for the evolutionary process that resulted in the fusion of these mating-type *loci* and the divergence in these chromosomal segments that have led to a suppression of recombination between the alleles [64]. Transcriptome analysis of *S. scitamineum* during *in vitro* growth and development *in planta* showed that mating-type genes are expressed in both conditions. The two mating-types were grown individually in order to obtain the RNAseq data *in vitro*, which suggests that contact is either not needed to favor gene expression of mating-type genes, or that a very short period of interaction is enough to induce gene expression, since cells were mixed immediately prior centrifugation and RNA extraction. The expression of mating-type genes at the *a* and *b* *loci* are induced by pheromone in *U. maydis*, that leads to amplification of the pheromone signal during the mating and to increased expression of *b* genes prior to fusion [72]. A single HMG box transcription factor, PrfI (pheromone response factor I), plays an important role in mediating this interaction between the *a* and *b* pathways. Prf1 protein binds specifically to pheromone response elements (PREs), which occur in clusters in the promoter regions in the vicinity of all genes in both *loci*[72]. Although in different numbers and orientations, PREs have been found close to the mating-type genes in *S. scitamineum* and other fungi studied, excepting *Malassezia* species to which so far no sexual cycle has been observed [73]. The *pfr1* gene is also expressed in all RNAseq experiments analyzed of *S. scitamineum*, suggesting that the same kind of regulation of *a* and *b loci* occurs in this species.

### Plant host-pathogen interaction transcriptome analysis

The combination of genome sequencing and transcriptome profiling is a proven approach to bring insights into pathogen mechanisms to invade host tissues, strategies of acquiring nutrients, avoid plant defense and to provoke disease symptoms. All these events are accomplished by a series of signals inducing a transcriptional reprogramming of fungal metabolism resulting in survival and dissemination within the host.

Our comparative transcriptome profiling showed that approximately 13.5% of *S. scitamineum* genes were either detected as differentially expressed *in planta* at 5 or 200 DAI. These genes are related to several metabolic processes important to fungal survival and protection in host tissues. One of these processes involves the chitin modification, mechanism that prevents the generation of elicitor active chitin oligomers which reveals the presence of the pathogen in the plant, triggering defense responses. The deacetylation of surface-exposed chitin into chitosan acts as a molecular disguise strategy [74–76]. Deacetylase is one of the most up-regulated gene at 5 DAI, indicating that *S. scitamineum* uses this strategy to dodge the plant defense in the early phases of fungal establishment. Que and collegues (2014) [77] determined the increase of chitinase gene expression in sugarcane transcriptome profiling of a resistance variety compared to susceptible plants.

The ability to pass through the plant cell wall by secreting a complex of extracellular cell wall degrading enzymes is evident in *S. scitamineum*. These enzymes are well known in filamentous plant pathogens and are necessary for entry into plant tissues [76, 78–81]. However, the amount of these enzymes may vary according to the fungal lifestyle. Biotrophic fungi can have fewer enzymes than hemibiotrophic and necrotrophic fungi [82]. In our study, the number of CAZymes (279) detected in the *S. scitamineum* genome is similar to other biotrophic fungus [83]. We found a small set of 54 cell wall degrading enzymes, some of which have been identified as up-regulated genes *in planta* at 5 and 200 DAI, such as those involved in cellulose, pectin and hemicellulose degradation.

Surpassing the cell barrier, pathogens have to breakdown proteins, sugars, lipids and nucleic acids [84]. Nineteen enzymes associated with digestion of proteins were detected among the secreted proteins. The transcriptome revealed that genes related to proteases were also up-regulated *in planta*. The fungal proteases may inhibit the activity of plant pathogenesis-related protein [85–87]. Another defense strategy potentially used by the fungus is the secretion of a cysteine-protease inhibitor to minimize the plant response, since the plant proteolytic machinery plays important roles in defense against pathogens [88].

Enzymes that target lipids, including lipases, triacyglycerol lipase and phospholipase were also found to have genes up-regulated at both time points analyzed. Lipases and phospholipases have been proposed to be essential not only for human pathogen virulence [89], but also for pathogens interacting with plants [90]. Eleven putative lipases were found in *U*. *maydis* potentially related to pathogenicity [91]. Lipase activities were associated with the fungal filamentous growth in vitro in the presence of lipids and also responsive to the known mating signaling pathways of the corn pathogen [91].

*S. scitamineum* seems to use another pathogen protective strategy to survive within sugarcane by the ability to detoxify the environment. Plants secrete various antimicrobial compounds into the apoplast to restrict pathogen growth. Examples are steroidal glycoalkaloids, such as saponin, and plant derived reactive oxygen species (ROS), which accumulate upon MAMP (microbe-associated molecular patterns) perception [92]. One of these detoxifying enzymes possibly used by *S. scitamineum* is related to pisatin demethylase, which was found up-regulated at 200 DAI. The pea pathogen *Fusarium oxysporum f. sp. pisi* is able to detoxify the phytoalexin pisatin, a substrate-inducible cytochrome P450, produced as a plant defense response [93]. Other genes related to cytochrome P450 and benzoate 4-monooxygenase are up-regulated at 200 DAI, which may indicate the need of detoxification *in planta*. These enzymes produce phenolic derivatives that are channeled to the b-ketoadipate pathway for aromatic compound degradation [94, 95]. Superoxide dismutase and catalases are also highly relevant to pathogenic fungal protection *in planta*[96, 97]. It has been reported that sugarcane resistant varieties produce an oxidative burst response upon smut infection [98]. These enzymes are involved in oxidative stress response against host superoxide radical (O_2_^-^) and hydrogen peroxide (H_2_O_2_), respectively. Their importance in the initial penetration is well documented [9, 99], but expression in the final stages (200 DAI) of smut sugarcane colonization in sugarcane may provide an additional protection against oxidative stress.

Of the differentially expressed genes at 5 DAI, nine have homologues in the PHI-base. Noteworthy are three up-regulated genes that code for sugar/glucose transporter and maltose permease, which in *U. maydis* mutants shows reduced virulence [100]. Among the differentially expressed genes at 200 DAI, 33 have homologues in the PHI-base. These genes are related to sugar, nicotinic acid, peptide transporters and the secreted proteins beta-glucosidase, lipase and aspartic protease. The sugar transporter coded by g1034_chr02_Ss is an ortholog of the *U. maydis* plasma membrane-localized sucrose transporter (Srt1), which is sucrose specific, and allows the direct utilization of sucrose without the production of extracellular monosaccharides known to elicit plant immune responses [100]. The presence of invertase is also indicative that sucrose breakdown is relevant for sporulation and whip development, leading support to the decrease of sucrose content in later stages of disease development [6]. The biotrophic interaction of *S. scitamineum* and sugarcane leads to increased invertase gene expression also by the plant, which is potentially to limit the sugar access to the pathogen, however the fungus present various sugar transporters that can increase the range of sugars and ways of intake this carbon source [101]. These mechanisms are probably among the ones that make this pathosystem very particular. Among other sugar transporter-encoding genes we found three quinate permease genes (g72_chr01_Ss; g1719_chr03_Ss and g2935_chr06_Ss) that were differentially expressed at 200 DAI. Quinate can serve as source of carbon to *M. oryzae* in early stages of rice infection [102]. *M oryzae* modulates expression of common genes related to the conversion between quinate and shikimate pathways by increasing quinate availability and decreasing products of the shikimate pathway, such as defensive phenylpropanoids. It is known that early sugarcane infection with *S. scitamineum* triggers expression of phenylpropanoid as a means of protection against fungal infection [77, 103]. In sugarcane it remains to be established whether quinate is available in the later stages of interaction, although these quinate assimilation genes up-regulated could be related to shunt of plant phenylpropanoid metabolism during smut whip emergence.

Laccase genes were also found to be differentially expressed in the fungus upon infection in sugarcane, revealing the potential of *S. scitamineum* for breaking down lignified tissues. Laccase is a polyphenol oxidase that catalyzes the reduction of O_2_ to H_2_O using a range of phenolic compounds as hydrogen donors, including lignin [104], that is the second most abundant constituent of the vascular plants cell wall, acting in cellulose protection towards hydrolytic microbial attack [105]. Sugarcane is known to respond to the presence of smut by modulating the expression of genes related to lignin pathways [98, 106, 107].

Our detailed annotation of the *S. scitamineum* genome revealed the presence of three genes encoding laccases. One of them, part of the described secretome, is encoded by the gene g1208_chr02_Ss, which is up-regulated in both 5 and 200 DAI, with values of Log_2_FoldChange of 6.56 and 7.59, respectively. Secreted laccases are related to lignin breakdown [108] and has great potential to be studied in numerous biotechnological applications, including those related to second generation of biofuels [97, 109, 110]. However, laccases can also be involved in various fungal physiological processes, for instance, the development of fruiting bodies [111] and spore pigmentation [112]. The transcriptome analysis results showed another laccase up-regulated at 200 DAI, which is not secreted (g4962_chr14_Ss, Log_2_FoldChange = 5.49). In this case this gene is possibly involved with pigment biosynthesis, since this stage of fungal development is characterized by intensive teliospore differentiation. Polyketide synthases encoding genes related to secondary metabolite production such as those involved in pigment biosynthesis were also found up-regulated at 200 DAI.

During the co-evolution of fungal plant pathogens and their hosts there has been a seesawing interplay between pathogen virulence and host resistance. Thus, to facilitate infection, plant pathogens secrete numerous effector proteins into the plant apoplast or cytosol [113]. Besides the strategies used to defend itself from plant immune system, *S. scitamineum* seems to have an arsenal of effectors that can potentially manipulate host metabolism [9, 114, 115]. *S. scitamineum* SSC39B secretome includes 70 proteins candidates for effectors, 51 annotated as either hypothetical or conserved hypothetical proteins. A similar number of candidates (43) was reported to occur in the 2014001 *S. scitamineum* strain [9]. Additionally, the authors used RT-qPCR analysis to reveal that 47% of these candidate genes were expressed in *S. scitamineum* in the early stages of the infection [9].

The transcriptomic data provided us with indications of *S. scitamineum* effectors that are transcriptionally active. Among them are the known effectors chorismate mutase, and salicylate hydroxylase [116], both involved in attenuating plant salicylic acid level reported in *U. maydis*[117], and Pep1 of *U. maydis*[118], an apoplastic inhibitor of host peroxidases [9, 119, 120]. As probable novel effectors used by *S. scitamineum* during sugarcane interaction we can highlight three genes (g2_chr01_Ss, g3890_chr10, and g1513_chr03_Ss) among the preferentially expressed in plant at 5 DAI, which encode to small secreted proteins of 236, 135 and 215 amino acids respectively. These genes have no identifiable conserved domains or any particular sequence feature. At 200 DAI the uncharacterized secreted proteins encoded by the highly expressed genes g3870_chr10_Ss, g488_chr01_Ss and g5684_chr17_Ss, are rich in glycine residues. In *M. oryzae*, members of the *pwl* gene family codify to small glycine-rich secreted proteins acting as Avrs conferring host specificity [121, 122].

A potential pathogenicity factor of the *S. scitamineum* is the most highly expressed genes of the secretome at 5 DAI (g3970_chr10_Ss). The encoded protein sequence presents several repeated motifs, such as “PQPQDGQ” residues represented seven times close to the N-terminal region and “PYGDKPNGDAENSDS” repeated eight times towards the C-terminal region. Its homologous in *U. maydis*, um03274, is expressed only in plant and not in axenic cultures [123] and in *S. scitamineum* the expression of this gene is low during in vitro growth. Sequences rich in proline and glutamine residues were found in the human fungal pathogen *Candida albicans*, where the 10-amino-acid long N-terminal repeat in the Hwp1p adhesin allows covalent cross-linking to host cells [124, 125]. All these genes of undetermined functions from *S. scitamineum* secretome, which are among the most expressed in plant, are good targets for experimental analyses to elucidate potential involvement in fungal growth and disease development.

In order to provide deeper analysis of *S. scitamineum* transcriptional profiles, we searched for the distribution of genes specifically expressed *in planta*, which allowed for the identification of 10 putative pathogenicity islands, a characteristic widespread in fungal pathogen genomes, which can harbor probable effectors. In the *U. maydis*, for instance were found 12 islands of genes encoding small secreted proteins with unknown function; most of them are co-regulated and induced in infected tissues, and deletion of individual islands can alter the pathogen virulence, leading in some cases to a complete lack of symptoms [7]. The evidence that some of them are only present in the *S. scitamineum* genome suggests its involvement in the sugarcane specificity, which is an important trait underlying the interaction of smuts with their hosts, but still poorly understood at the molecular level. Despite being phylogenetically close, the smut fungi infect different members of the Poaceae, and vary in their mode of plant colonization and symptom development. Searching for species-specific genes is a promising strategy to identify genes involved in host-specific adaptations [51, 126]. An important feature found in four of the predicted islands (chromosomes 2, 6, 10 and 11) is the presence of repetitive elements, that have been viewed as drivers of genome evolution by promoting genome rearrangements and possible gene regulation [127], and this can be related to the smut fungus adaptability towards sugarcane colonization.

### Conclusions

The *de novo* complete genome assembly allowed us the determination of subtelomeric regions, mating-type *loci*, repetitive elements and the sequence and annotation of mtDNA of *S. scitamineum*. The mtDNA with 88 kb in length is within the size range expected for members of the Ustiloginales and the genome is rich in potential mobile introns with some evidence that duplications may have been generated by the activity of intron encoded homing endonucleases. Comparisons to other smut genome sequences revealed that chromosomal reorganizations related to the mating *loci* and details of the sequences linking both *loci* may be used in further evolutionary studies. The combination of transcriptomic data obtained in different phases of the fungal life cycle disclosed modulation of gene expression revealing that *S. scitamineum* uses common strategies to survive within sugarcane but also uncovered novelties that are associated to the specific interaction with sugarcane. The onset of infection is similar to those of other smut interactions. For instance, orthologues of *U. maydis* known virulence factors were found to be expressed in *S. scitamineum* during infection phase by means of attenuation of the salicylate mediated defense response. New candidate effectors have been identified and are organized in 10 genomic islands which are expressed only *in planta*, some of which *S. scitamineum* specific. Plant cell wall degrading enzymes, proteases and lipases are up-regulated potentially associated to the entry of the pathogen. Fungal defense responses were also a common strategy regarding the interaction with sugarcane. The fungus accumulates a series of transcripts encoding proteins to survive ROS and other toxins potentially produced by the plant. To avoid recognition by the host plant *S. scitamineum* probably uses chitin modifications. During the development of the whip and sporogenesis the presence of transcripts related to detoxification of plant metabolites as well as ROS are evident. The fungus metabolism shifts to the accumulation of sugar transporters and invertase transcripts, to use sucrose directly and/or sucrose monomers derivatives. Increased laccase activities both extracellular and non-secreted were detected. Laccases are regarded as potentially involved in lignin degradation and to pigment biosynthesis. This study suggests a new promising research aiming at the biotechnological use of *S.scitamineum* as well as provides valuable targets for experimental studies to confirm pathogen genes involved parasite/host interaction, with perspectives for applications in disease management and sugarcane breeding.

## Database accession number

The complete genome sequence of the *S. scitamineum* SSC39B has been submitted at DDBJ/EMBL/GenBank under the BioProject number: PRJNA275631

The reads used in the RNAseq analysis has been submitted at DDBJ/EMBL/GenBank under the BioProject number: PRJNA275890

The complete genome sequence of the *S. scitamineum* BSES15 and BSES17 has been submitted the BioProject number: PRJEB5169

## Supporting Information

S1 File General analysis of *S. scitamineum* genome (Excel file format). Chromosomes size, coordinates of telomeric regions and pattern found by Tandem Repeat Database **(Table A)**. Circos of the alignment between the tree small contigs and the other chromosomes (a) and between other strains of *S. scitamineum* **(Figure A)**. Pulse Field Gel Hibridizations **(Figure B)**. Predicted tRNAs obtained by using tRNAscan-SE **(Table B)**. Genes coordinates, best hit of each *S. scitamineum* protein against Uniref50, presence of signal peptide, transmembrane domain, GPI anchor site and RNAseq length coverage and BLASTp against proteins of *S. reilianum* **Table C)**. Gene length distribution in *S. scitamineum genome* **(Figure C)**. Annotation of subtelomeric genes **(Table D)**. Repetitive elements detected using RECON for de novo identification, REPBASE and RepeatMasker **(Table E)**. dbCAN domains on CAZymes **(Table F)**. Alignments against the reference proteins from PHI-base **(Table G)**.
(XLS)

S2 File Blast2GO analysis (Excel file format). Protein annotation of *S. scitamineum* genome using Blast2GO **(Table A)**. Distribution of GO terms of predicted *S. scitamineum* proteins **(Table B)**. List of best hit species of predicted protein sequences of *S. scitamineum* as defined by Blast2GO analysis **(Table C)**.
(XLS)

S3 File Chromosomes comparison of *S. scitamineum* SSC39 and *S. reilianum* (Zip with figures). Figures produced using Circos software to illustrate chromosomes alignments between these two close related species.
(ZIP)

S4 File Mating-type analysis (PDF file format). Percentage of identity of mating-type proteins and the average of predicted proteins from whole genome between *S. scitamineum* and others smut fungi **(Table A)**. Unrooted consensus phylogenetic tree for the mating-type proteins from smut related species **(Figure A)**. Genomic context of mating-type genes from smut related species **(Figure B)**.
(PDF)

S5 File Mitochondrial genome analysis (Excel file format). Table with mitochondrial gene annotations and manual curation **(Table A)**. Feature table of the mitochondria **(Table B)**.
(XLS)

S6 File OrthoMCL analysis (Excel file format). Co-orthologs groups with their respective members **(Table A)**. Four-way-Venn-diagram showing the distribution of orthologous protein clusters among smuts species **(Figure A)**. Unique orthologs groups and theirs predicted functions **(Table B)**. Singletons identifiers (proteins not assigned to any OrthoMCL cluster) **(Table C)**. Single-copy genes in each group of orthologs **(Table D)**. Putative effectors unique of *S. scitamineum* (differentially or exclusively) expressed*in planta* **(Table E)**.
(XLS)

S7 File Gene Ontology analysis of *S. scitamineum* secretome (PDF file format). Graphs with GO terms enriched in the *S. scitamineum* secretome **(Figure A)**.
(PDF)

S8 File Transcriptome data analysis (Excel file format). Number of reads mapped in transcriptome analysis obtained with CLC Genomics Workbench analysis (100% identity, 98% coverage) **(Table A)**. Genes preferentially expressed *in vitro*, 5 DAI and 200 DAI. Expression values were defined by normalization using scaling approach and CDS size **(Table B)**. Genes differentially expressed at 5 DAI in comparison with transcriptional profiles obtained in YM culture medium fungal axenic growth. Were considered differentially expressed genes those with FDR ≤ 0.01 in Baggerley’s test and Log2(Fold Change) ≤ -2 or ≥ 2 **(Table C)**. Genes differentially expressed at 200 DAI as assumed in 5 DAI analysis **(Table D)**. GO terms enrichment of differentially expressed genes using p-value ≤ 0.05 performed in Blast2GO software **(Table E)**. Genes expressed exclusively in plant in comparison with genes expressed during axenic growth in YM medium **(Table F)**.
(XLS)
