# Supplementary figures and images for: Complete Genome Sequence of Sporisorium scitamineum and Biotrophic Interaction Transcriptome with Sugarcane
Source: PLoS One. 2015 Jun 12;10(6):e0129318. doi: 10.1371/journal.pone.0129318 (PMC4466345; doi:10.1371/journal.pone.0129318)

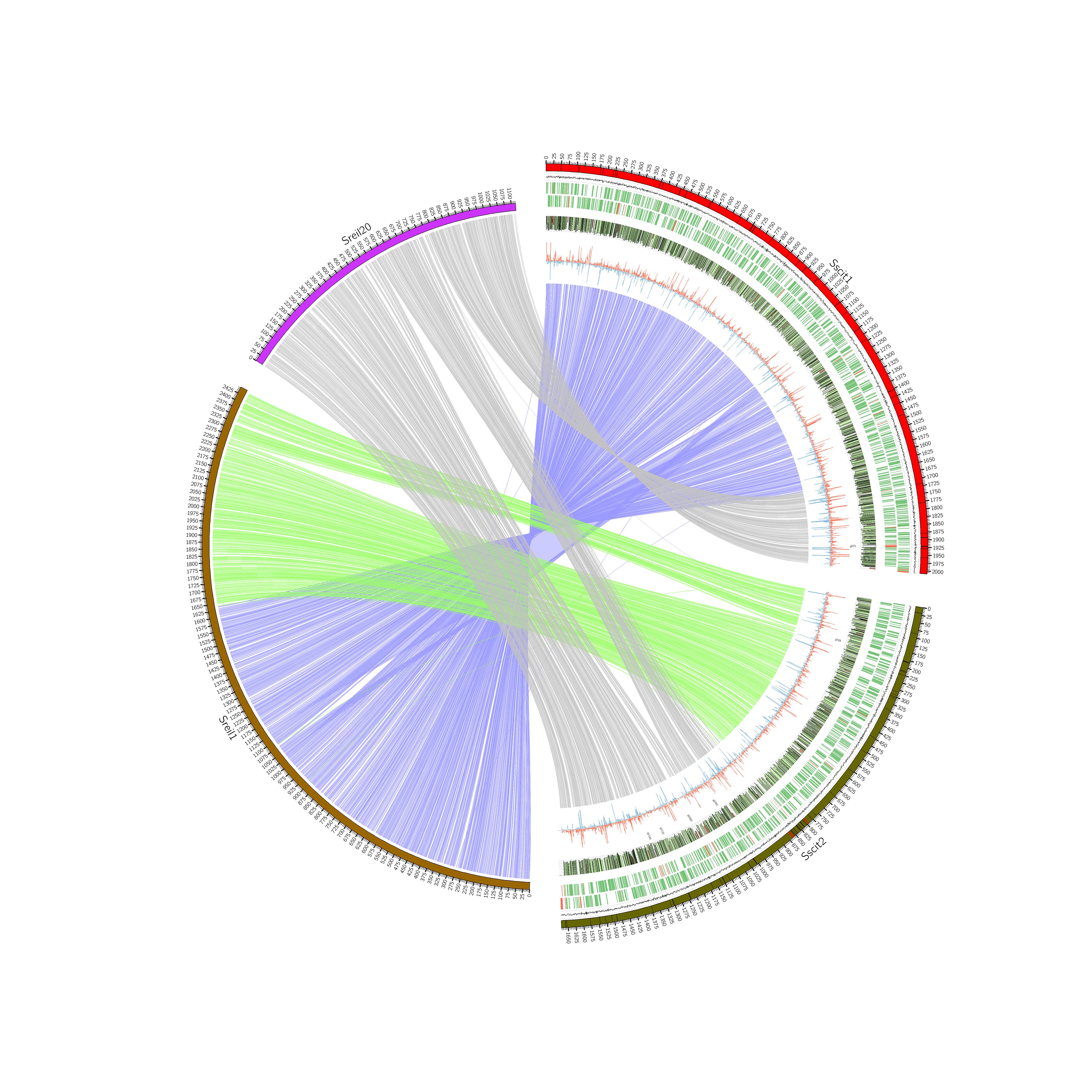

Supplement: S3 File — Figures produced using Circos software to illustrate chromosomes alignments between these two close related species. (ZIP) [file pone.0129318.s003.zip › chromo_1_2.jpg]

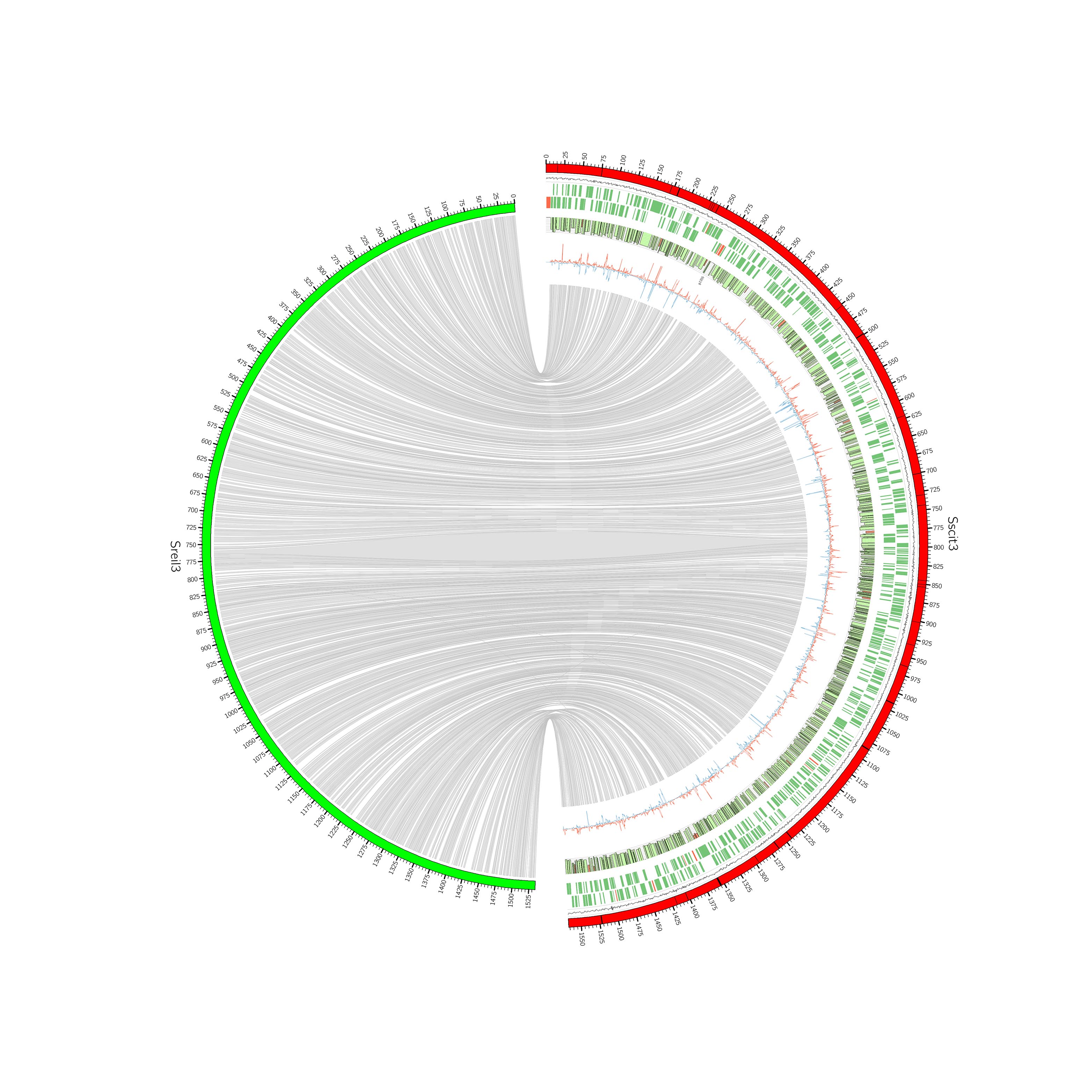

Supplement: S3 File — Figures produced using Circos software to illustrate chromosomes alignments between these two close related species. (ZIP) [file pone.0129318.s003.zip › chromo_3.jpg]

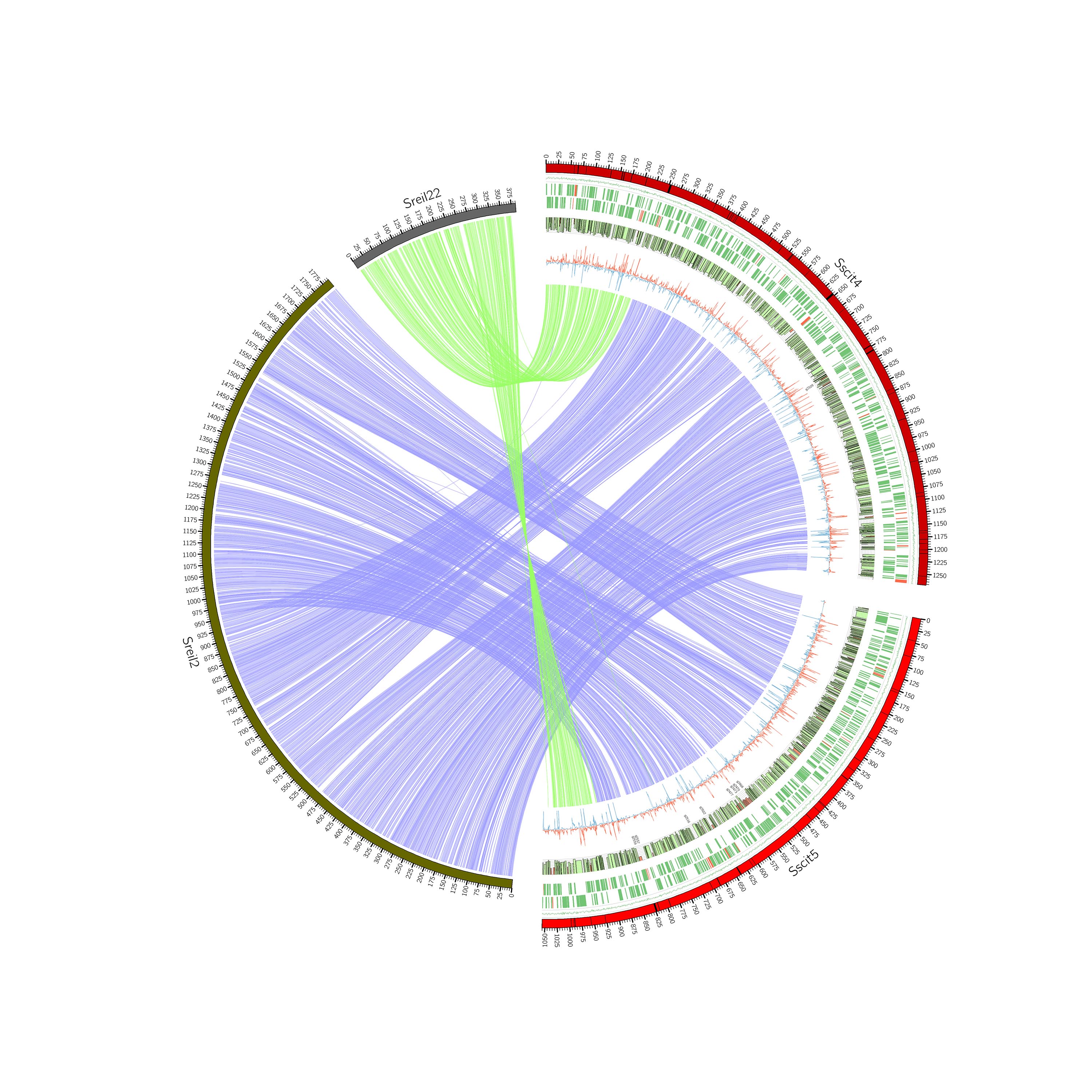

Supplement: S3 File — Figures produced using Circos software to illustrate chromosomes alignments between these two close related species. (ZIP) [file pone.0129318.s003.zip › chromo_4_5_eff.jpg]

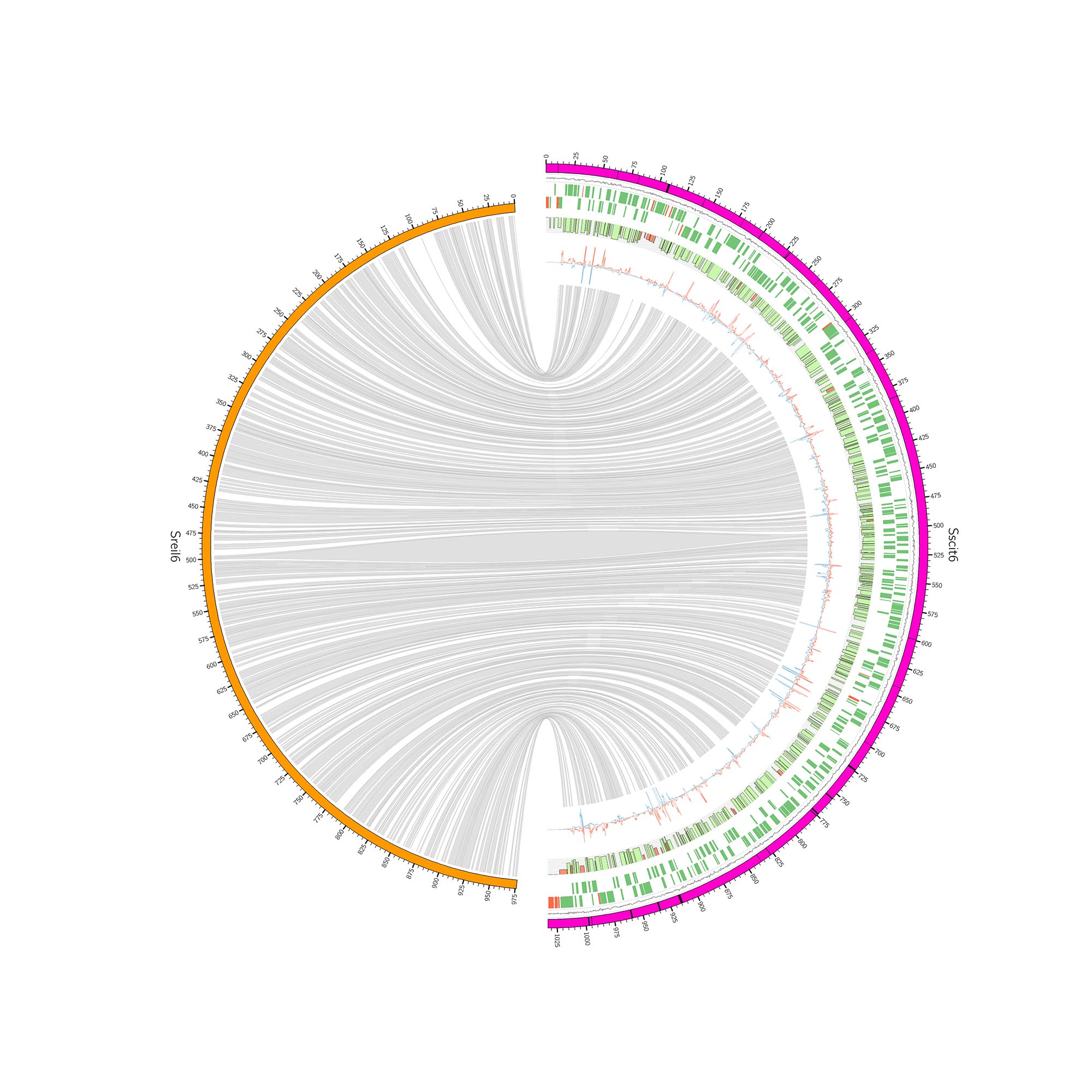

Supplement: S3 File — Figures produced using Circos software to illustrate chromosomes alignments between these two close related species. (ZIP) [file pone.0129318.s003.zip › chromo_6.jpg]

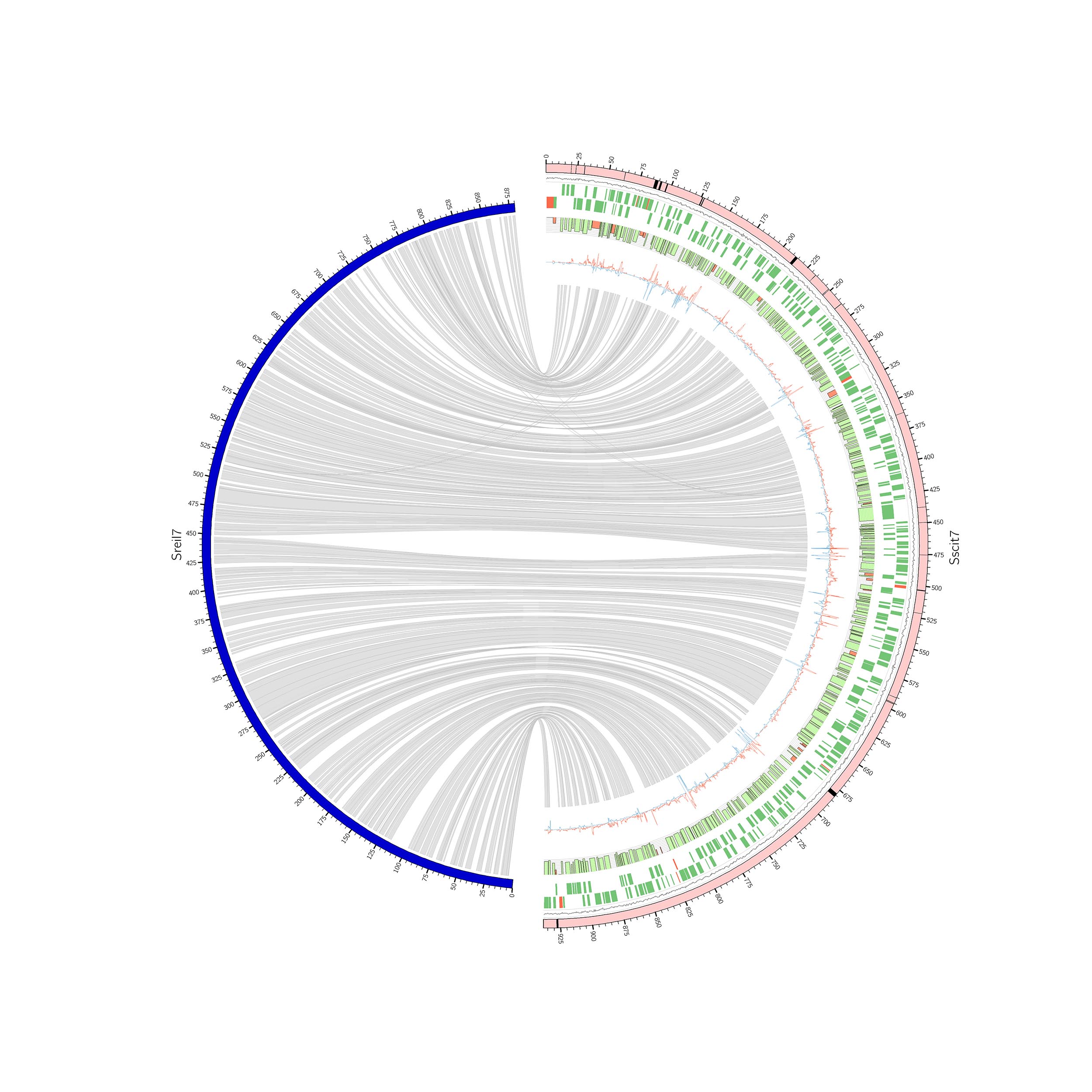

Supplement: S3 File — Figures produced using Circos software to illustrate chromosomes alignments between these two close related species. (ZIP) [file pone.0129318.s003.zip › chromo_7.jpg]

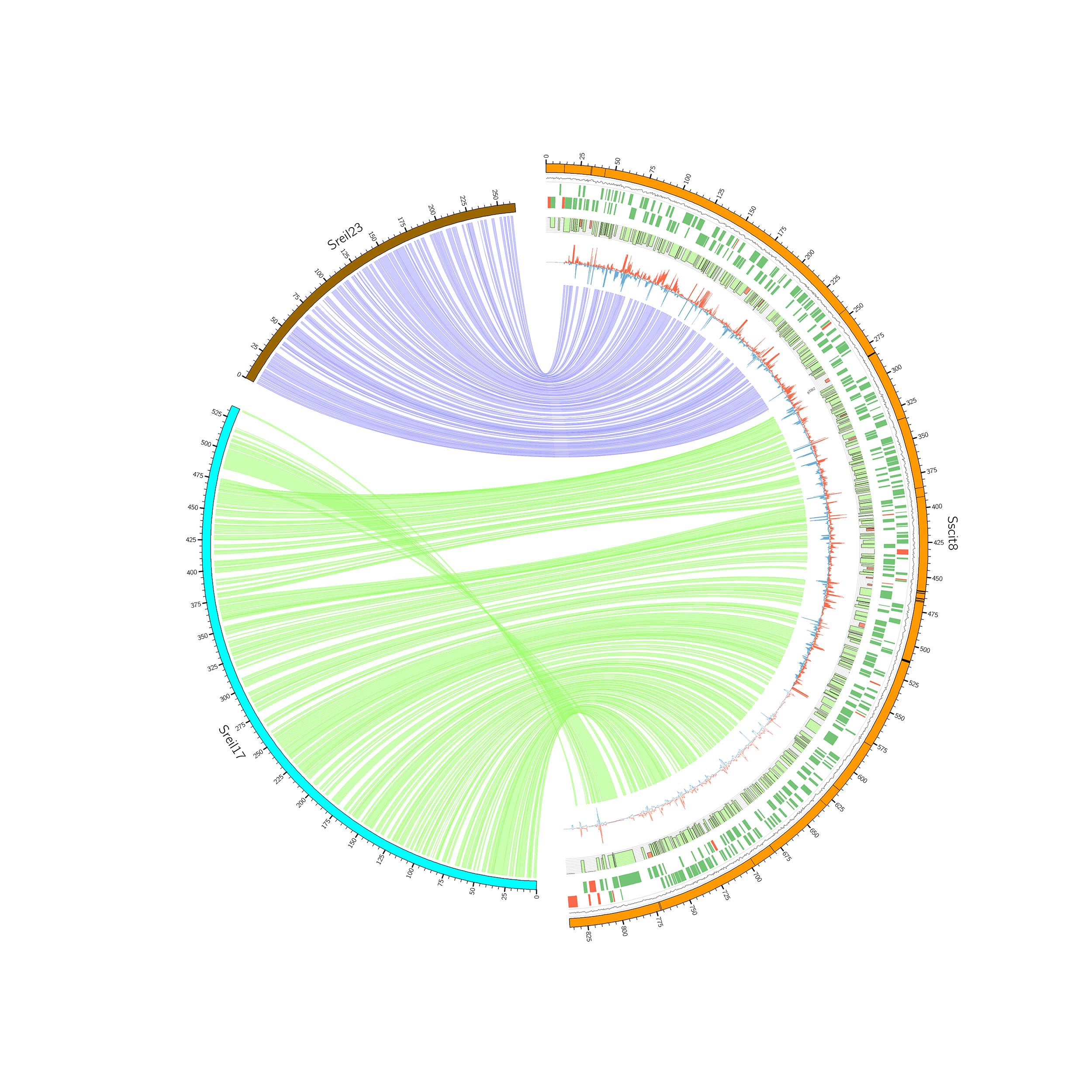

Supplement: S3 File — Figures produced using Circos software to illustrate chromosomes alignments between these two close related species. (ZIP) [file pone.0129318.s003.zip › chromo_8.jpg]

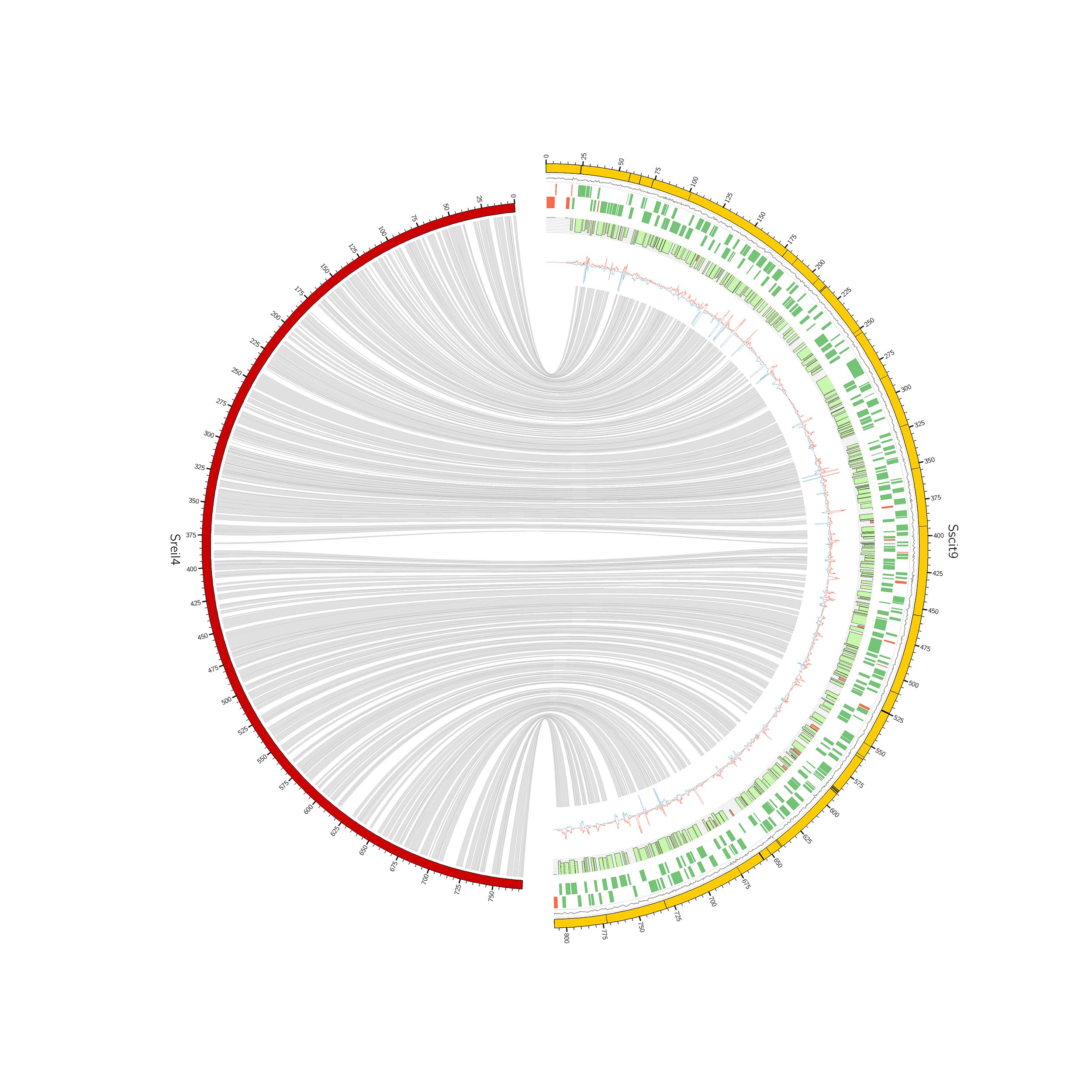

Supplement: S3 File — Figures produced using Circos software to illustrate chromosomes alignments between these two close related species. (ZIP) [file pone.0129318.s003.zip › chromo_9.jpg]

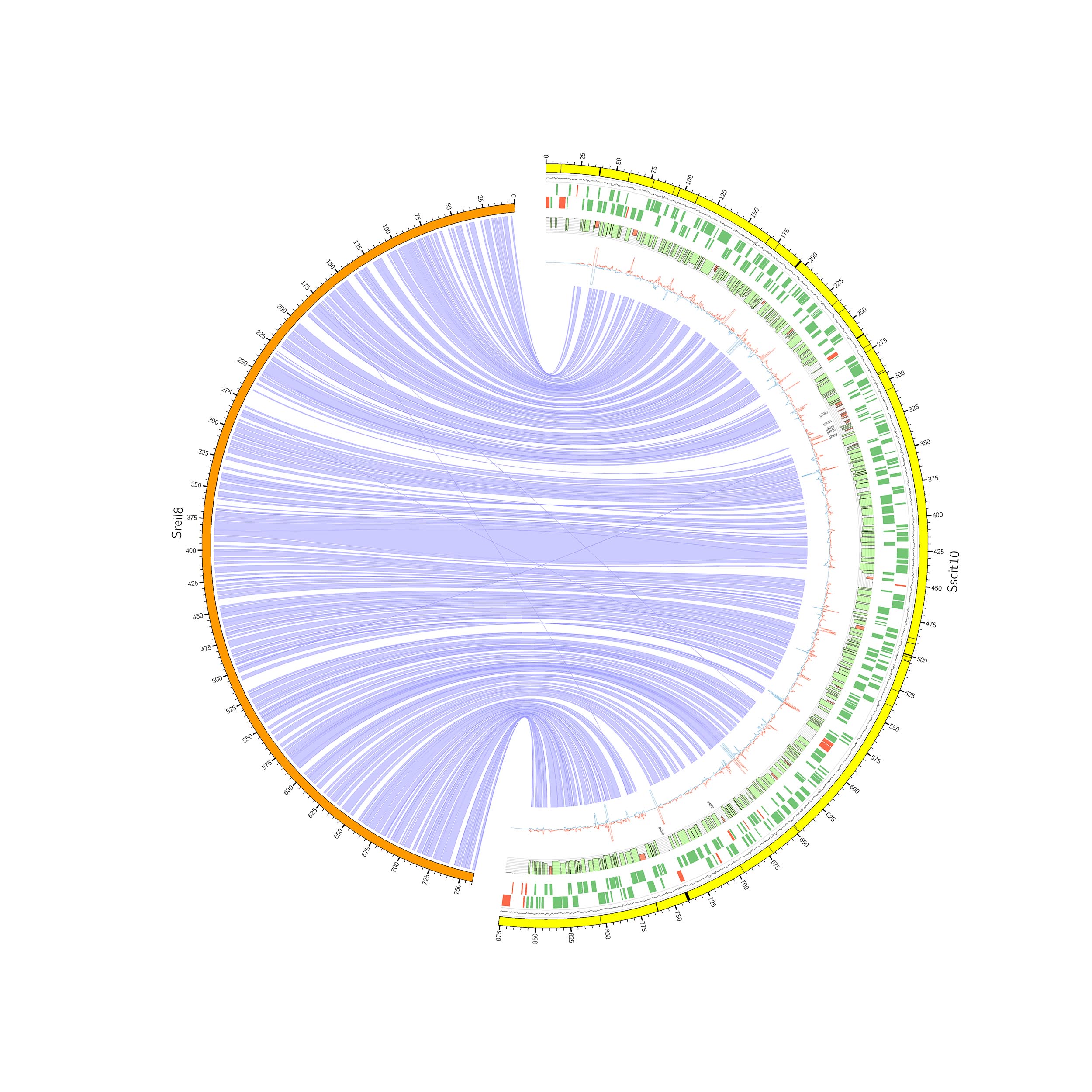

Supplement: S3 File — Figures produced using Circos software to illustrate chromosomes alignments between these two close related species. (ZIP) [file pone.0129318.s003.zip › chromo_10.jpg]

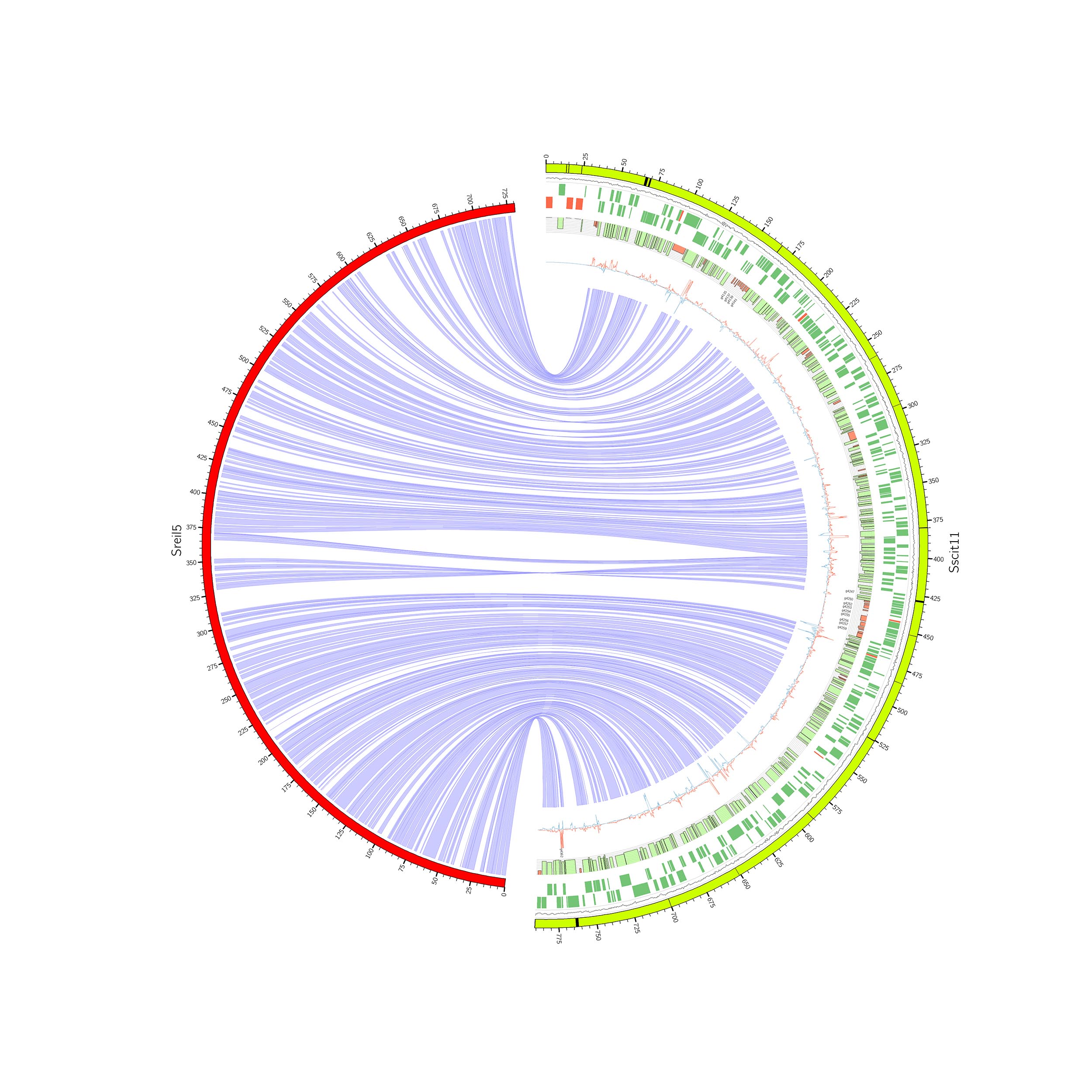

Supplement: S3 File — Figures produced using Circos software to illustrate chromosomes alignments between these two close related species. (ZIP) [file pone.0129318.s003.zip › chromo_11.jpg]

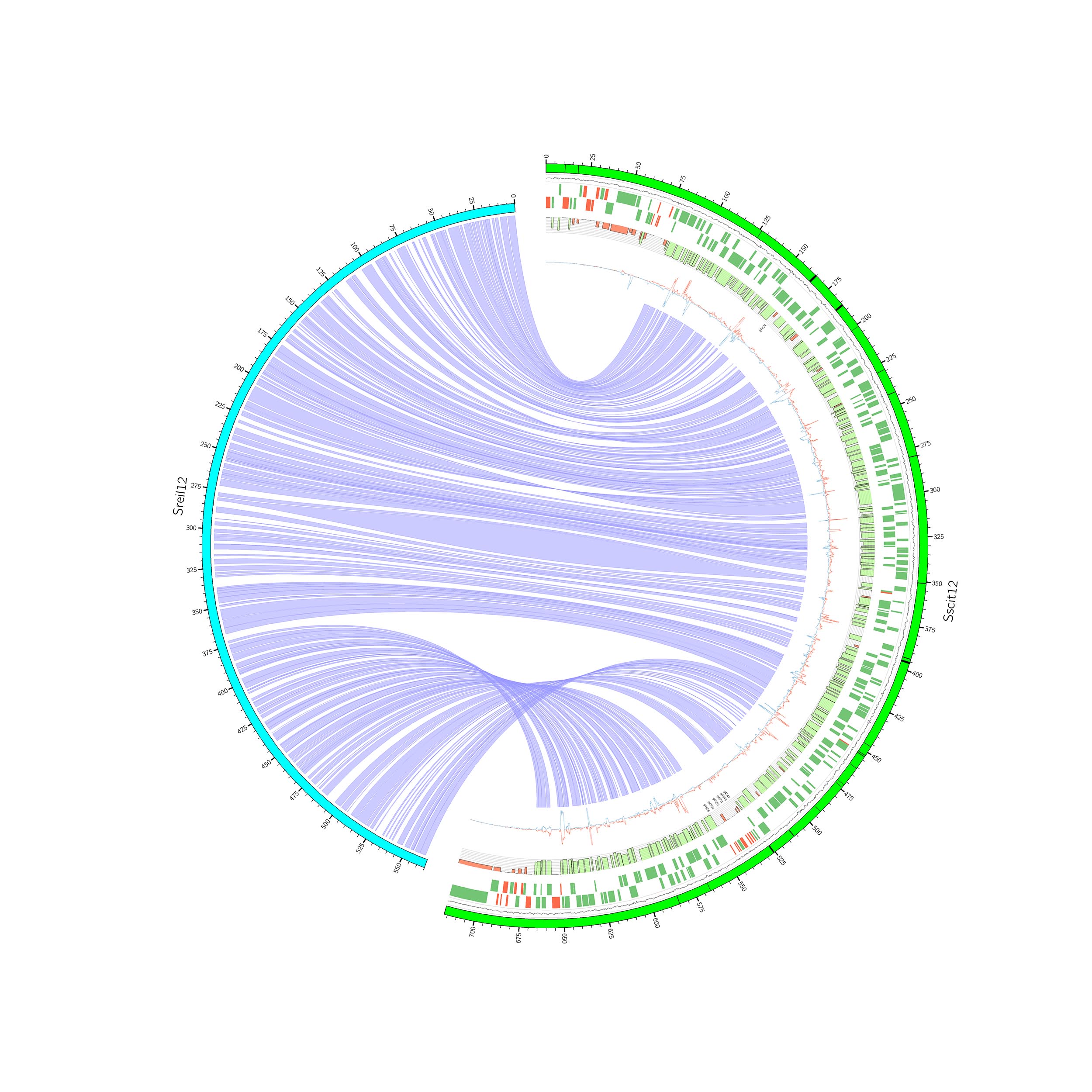

Supplement: S3 File — Figures produced using Circos software to illustrate chromosomes alignments between these two close related species. (ZIP) [file pone.0129318.s003.zip › chromo_12.jpg]

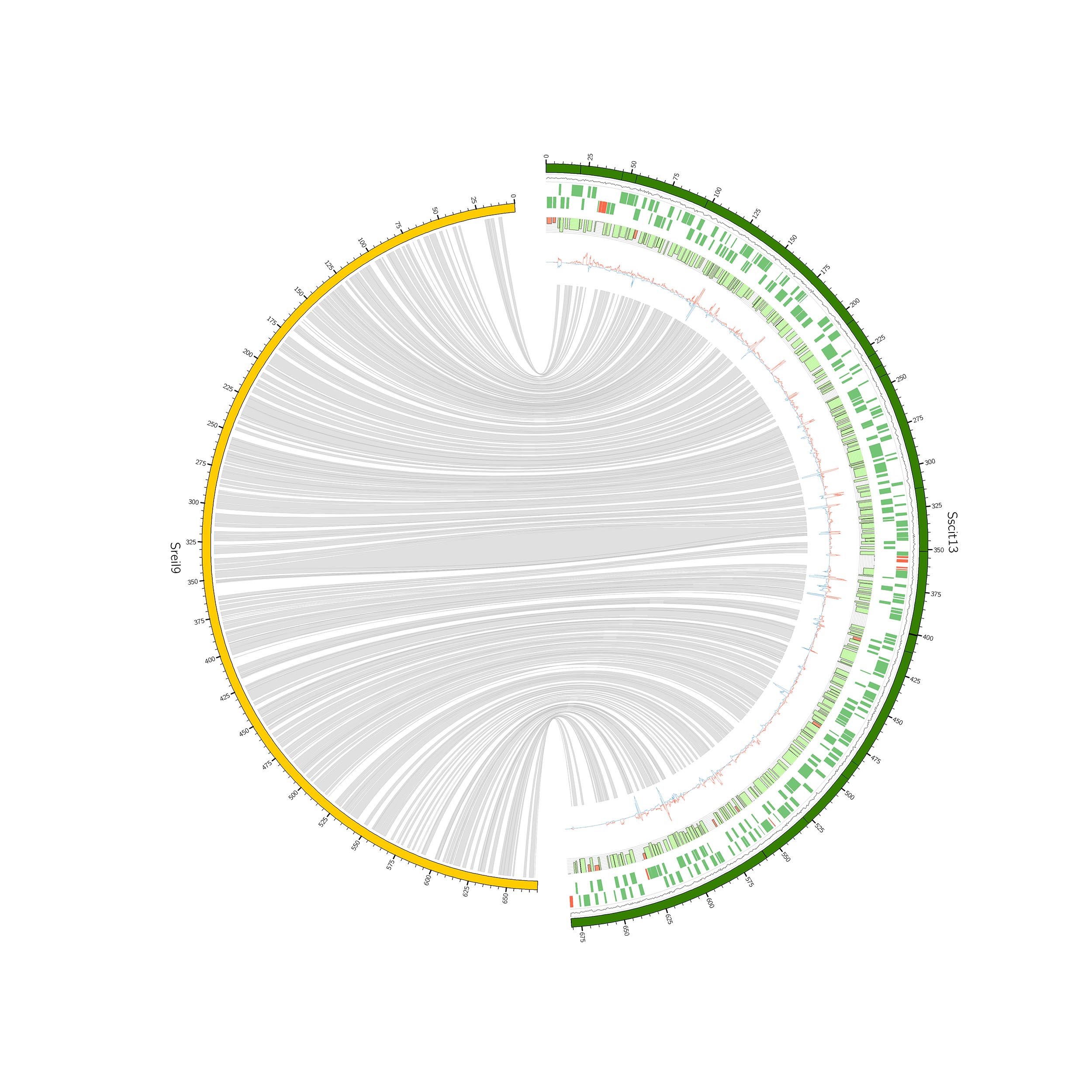

Supplement: S3 File — Figures produced using Circos software to illustrate chromosomes alignments between these two close related species. (ZIP) [file pone.0129318.s003.zip › chromo_13.jpg]

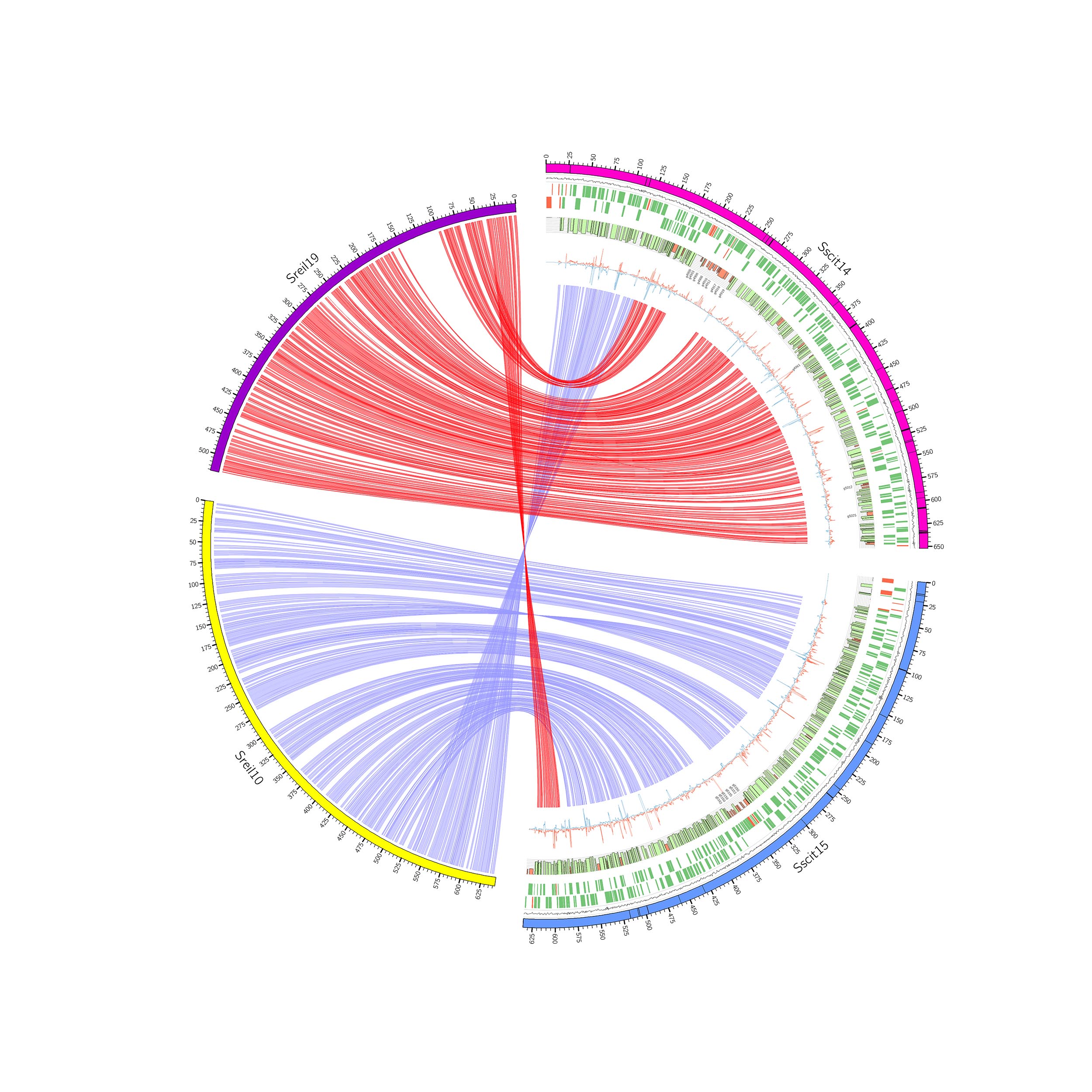

Supplement: S3 File — Figures produced using Circos software to illustrate chromosomes alignments between these two close related species. (ZIP) [file pone.0129318.s003.zip › chromo_14_15.jpg]

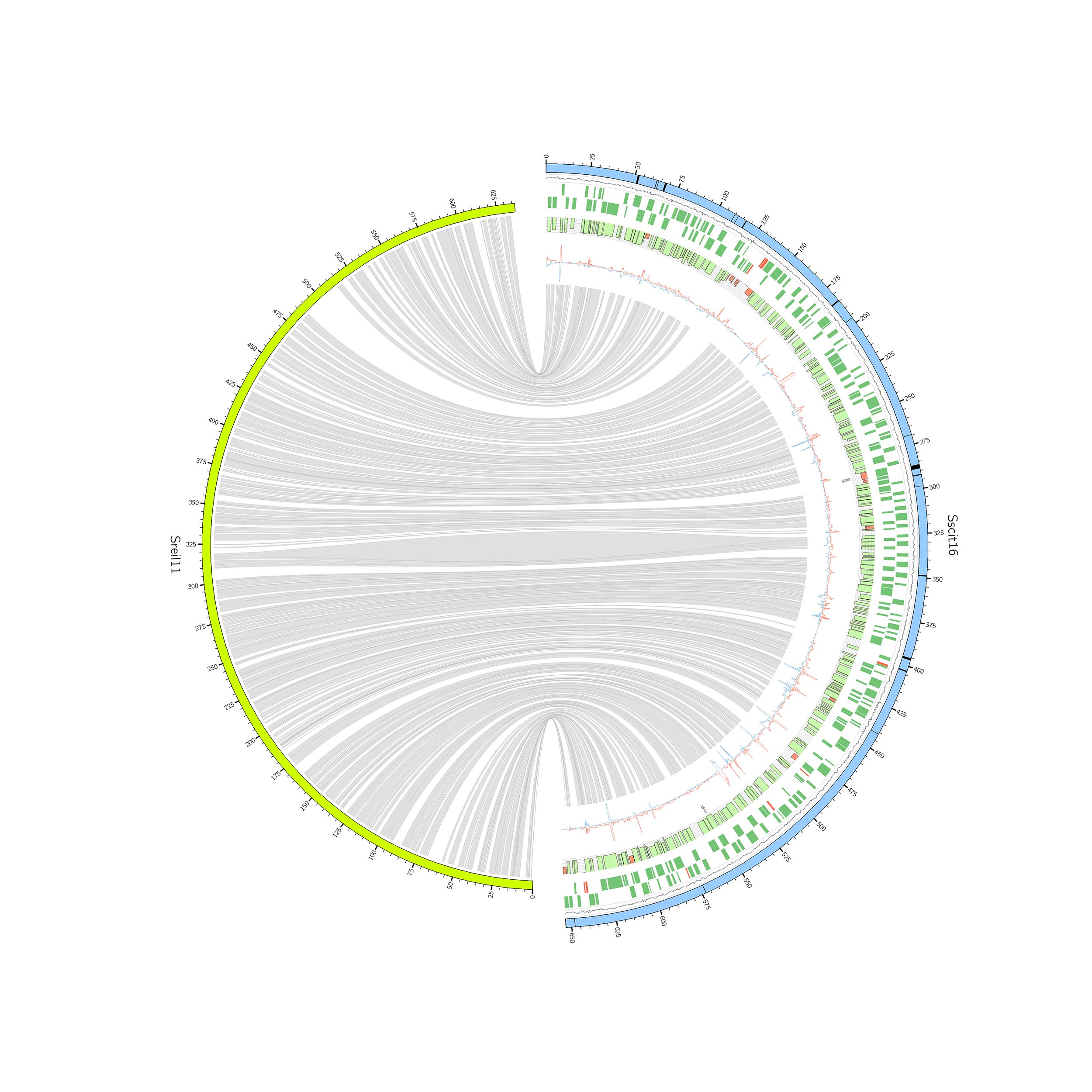

Supplement: S3 File — Figures produced using Circos software to illustrate chromosomes alignments between these two close related species. (ZIP) [file pone.0129318.s003.zip › chromo_16.jpg]

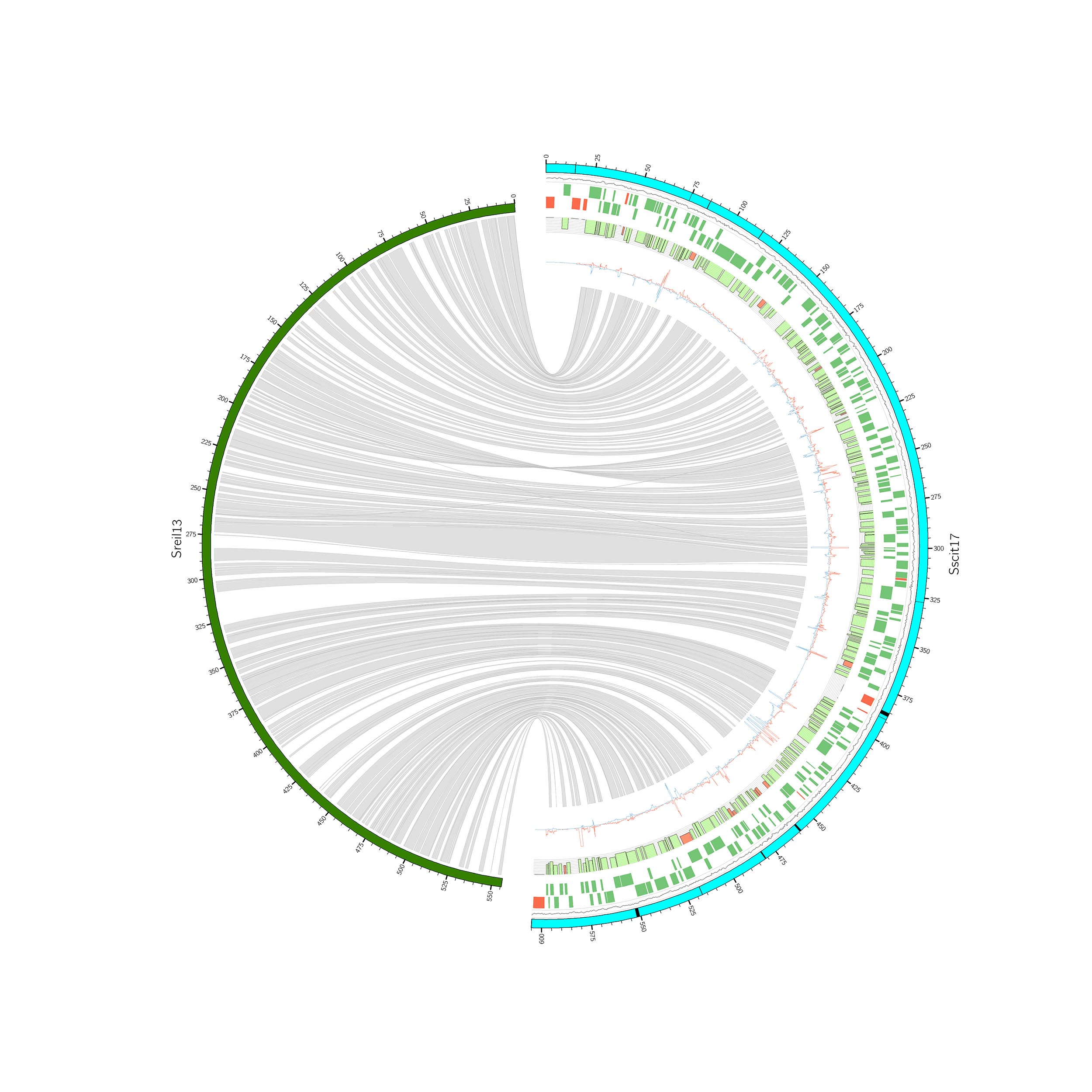

Supplement: S3 File — Figures produced using Circos software to illustrate chromosomes alignments between these two close related species. (ZIP) [file pone.0129318.s003.zip › chromo_17.jpg]

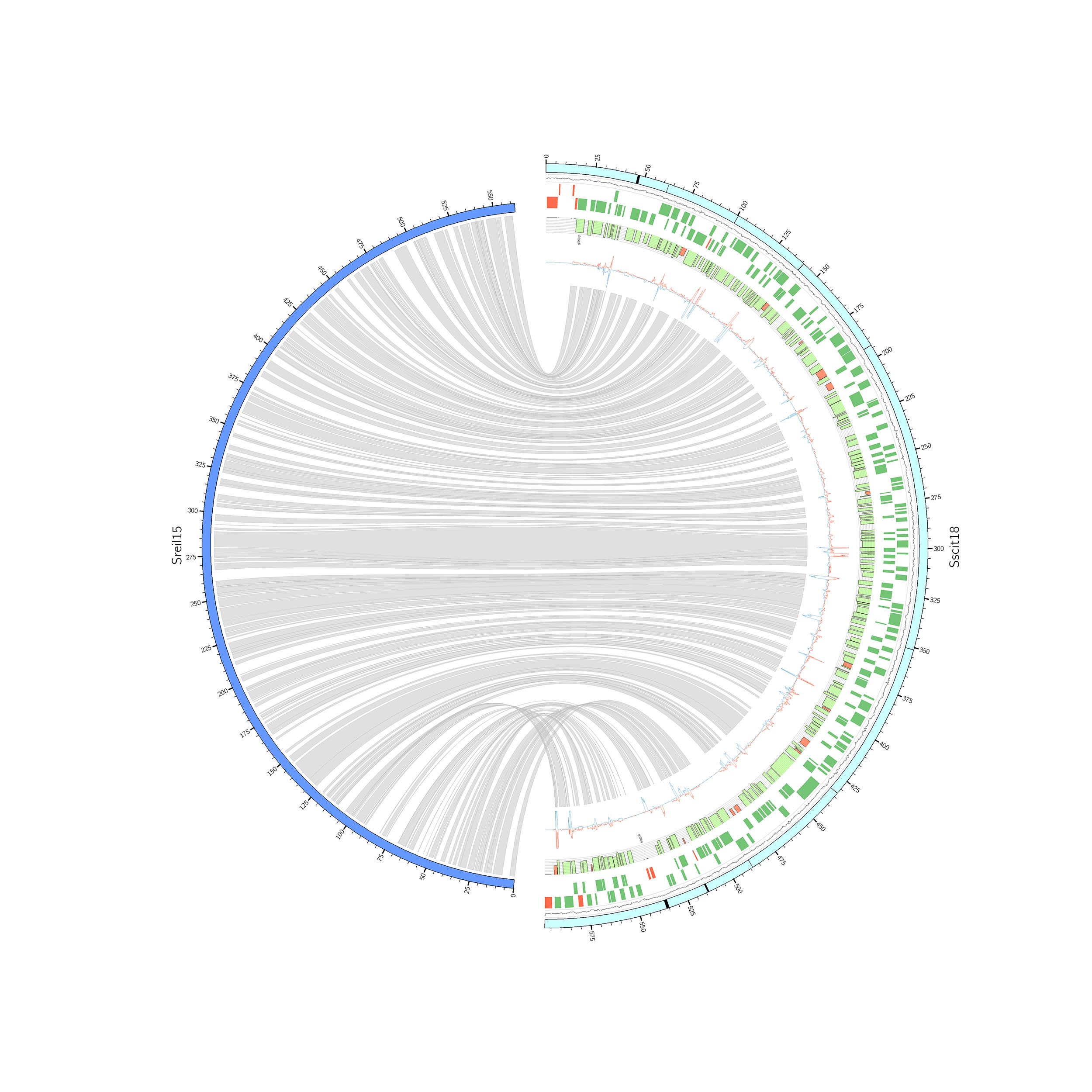

Supplement: S3 File — Figures produced using Circos software to illustrate chromosomes alignments between these two close related species. (ZIP) [file pone.0129318.s003.zip › chromo_18.jpg]

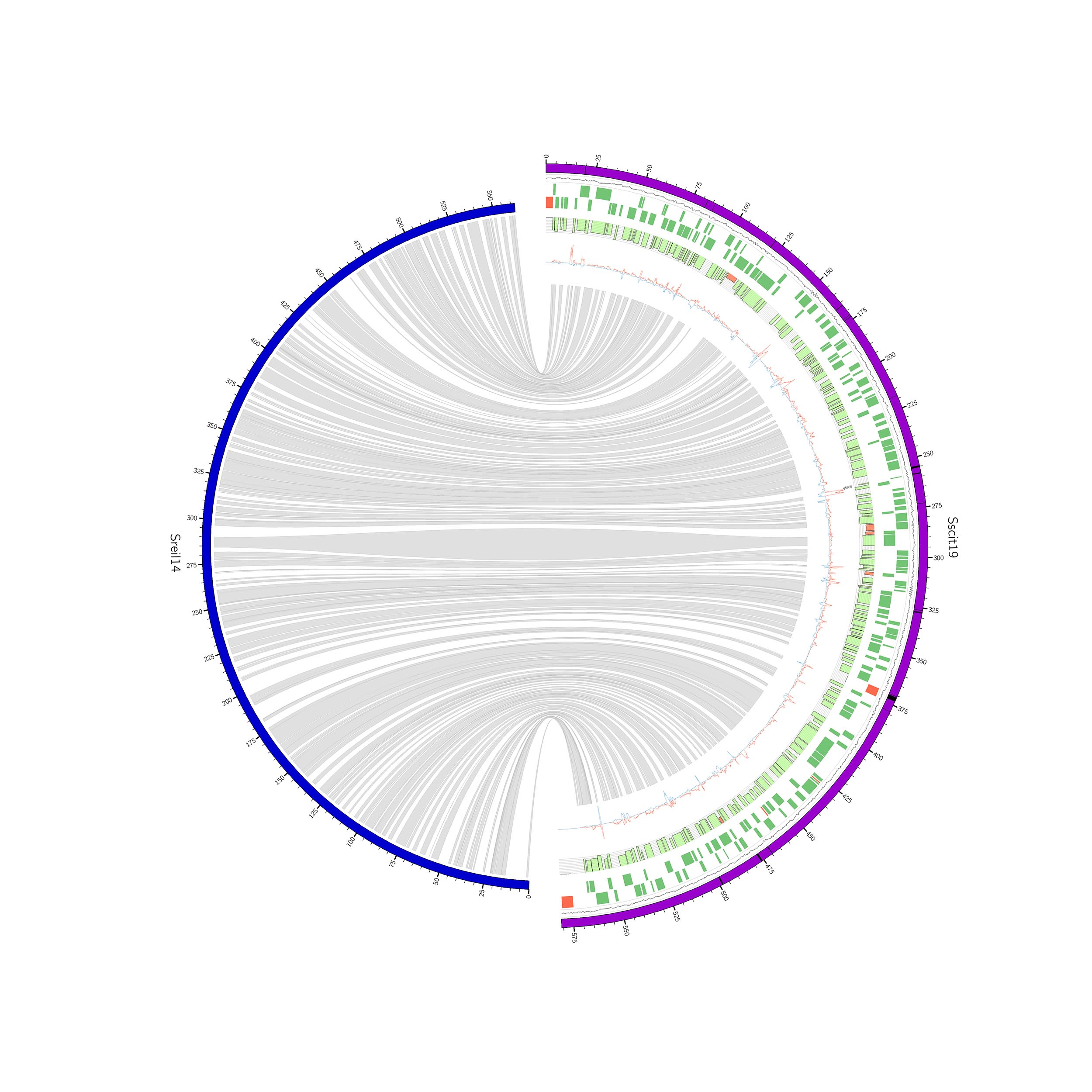

Supplement: S3 File — Figures produced using Circos software to illustrate chromosomes alignments between these two close related species. (ZIP) [file pone.0129318.s003.zip › chromo_19.jpg]

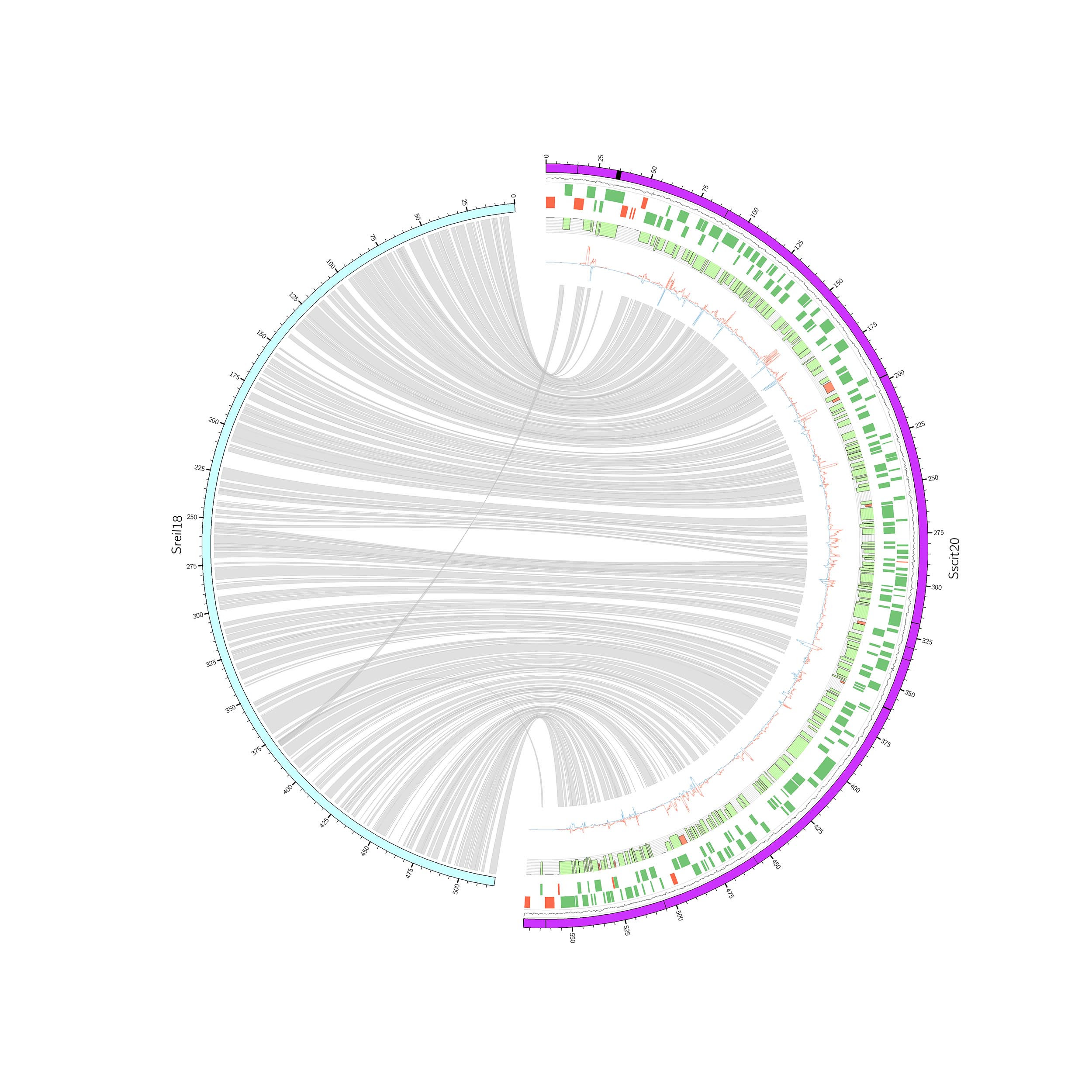

Supplement: S3 File — Figures produced using Circos software to illustrate chromosomes alignments between these two close related species. (ZIP) [file pone.0129318.s003.zip › chromo_20.jpg]

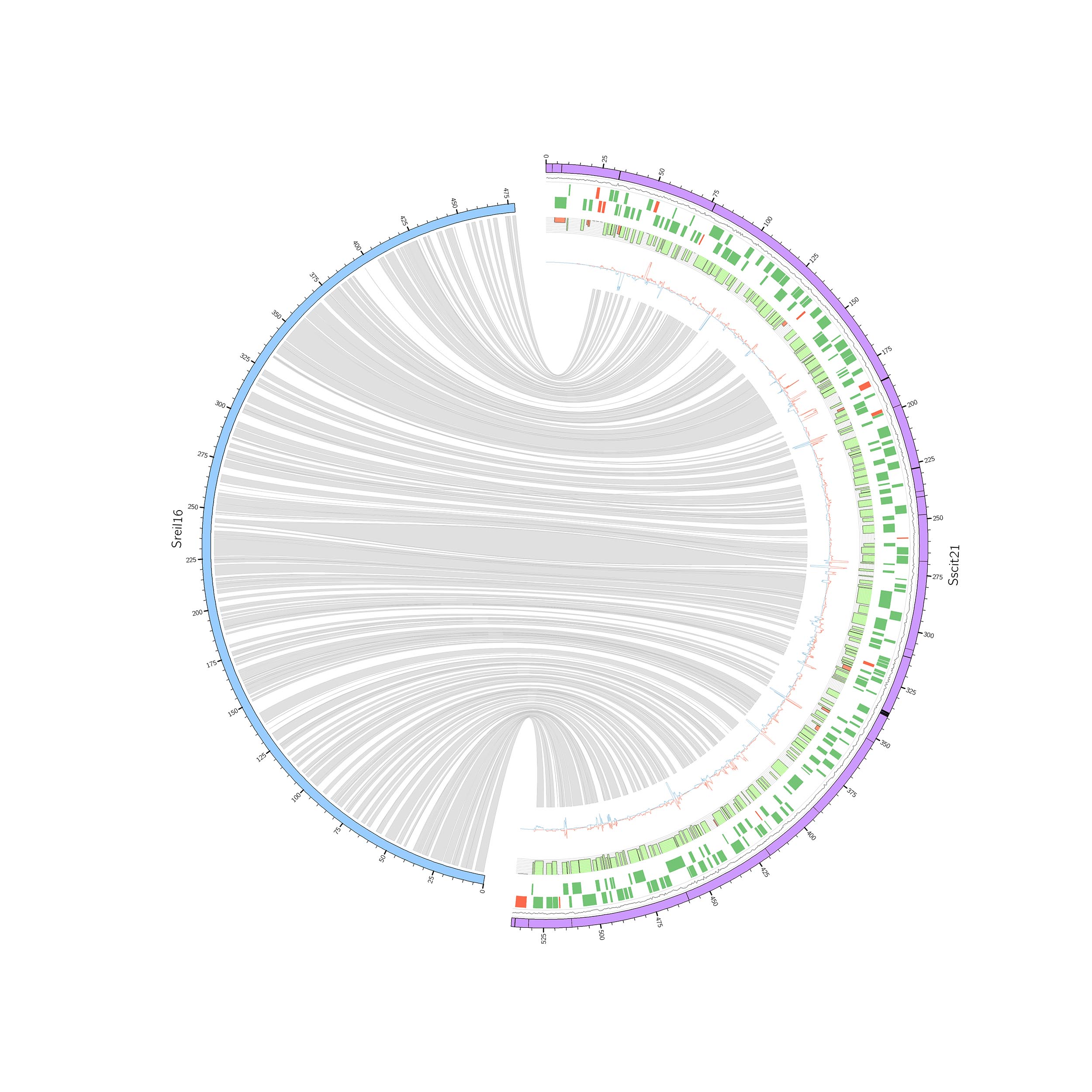

Supplement: S3 File — Figures produced using Circos software to illustrate chromosomes alignments between these two close related species. (ZIP) [file pone.0129318.s003.zip › chromo_21.jpg]

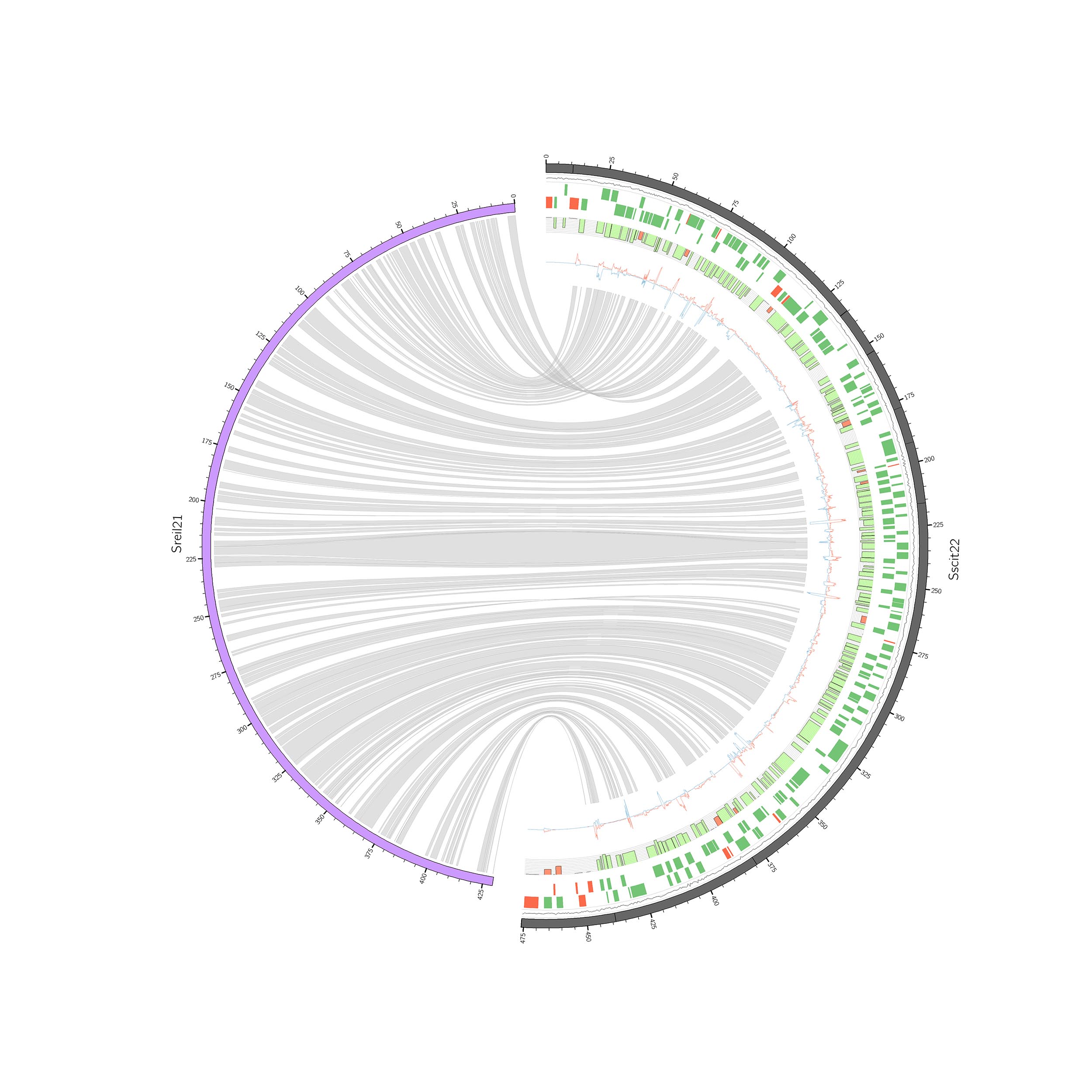

Supplement: S3 File — Figures produced using Circos software to illustrate chromosomes alignments between these two close related species. (ZIP) [file pone.0129318.s003.zip › chromo_22.jpg]
