# Supplementary material for: Complete Genome Sequence of Sporisorium scitamineum and Biotrophic Interaction Transcriptome with Sugarcane
Source: PLoS One. 2015 Jun 12;10(6):e0129318. doi: 10.1371/journal.pone.0129318 (PMC4466345; doi:10.1371/journal.pone.0129318)
Supplement: S4 File — Percentage of identity of mating-type proteins and the average of predicted proteins from whole genome between S. scitamineum and others smut fungi (Table A). Unrooted consensus phylogenetic tree for the mating-type proteins from smut related species (Figure A). Genomic context of mating-type genes from smut related species (Figure B). (PDF) [file pone.0129318.s004.pdf]

S4 – Supporting Information
Mating-type analysis

Table A. Percentage of identity of mating-type proteins andthe average of predicted proteins from whole genome between *S. scitamineum* and others smut fungi.

***S. scitamineum* proteins**	**Percentage identity (BLASTp e-value ≤ 1 x e-14)**		
	S. reilianum	***U. maydis***	***U. hordei***
Average of all predicted proteins	82.5%	75.4 %	72.4%
bE1	68% (bE2)	53% (bE1)	50% (bE1)
bW1	63% (bW2)	43% (bW1)	41% (bW1)
pra1	28% (pra2)	72% (pra1)	69% (pra1)
mfa1.2	Ns* (mfa2.1)<br>Ns*(mfa2.3)	73% (mfa1.2)	Ns* (mfa1.2)
mfa1.3	Ns* (mfa2.1)<br>66% (mfa2.3)	Ns* (mfa1.2)	Ns* (mfa1.2)

NOTE - The mating-type proteins compared here were those present in the haploid genome sequenced of each smut fungi to allow the comparison with the average identity of proteins from the whole genome.
*Ns - No significant

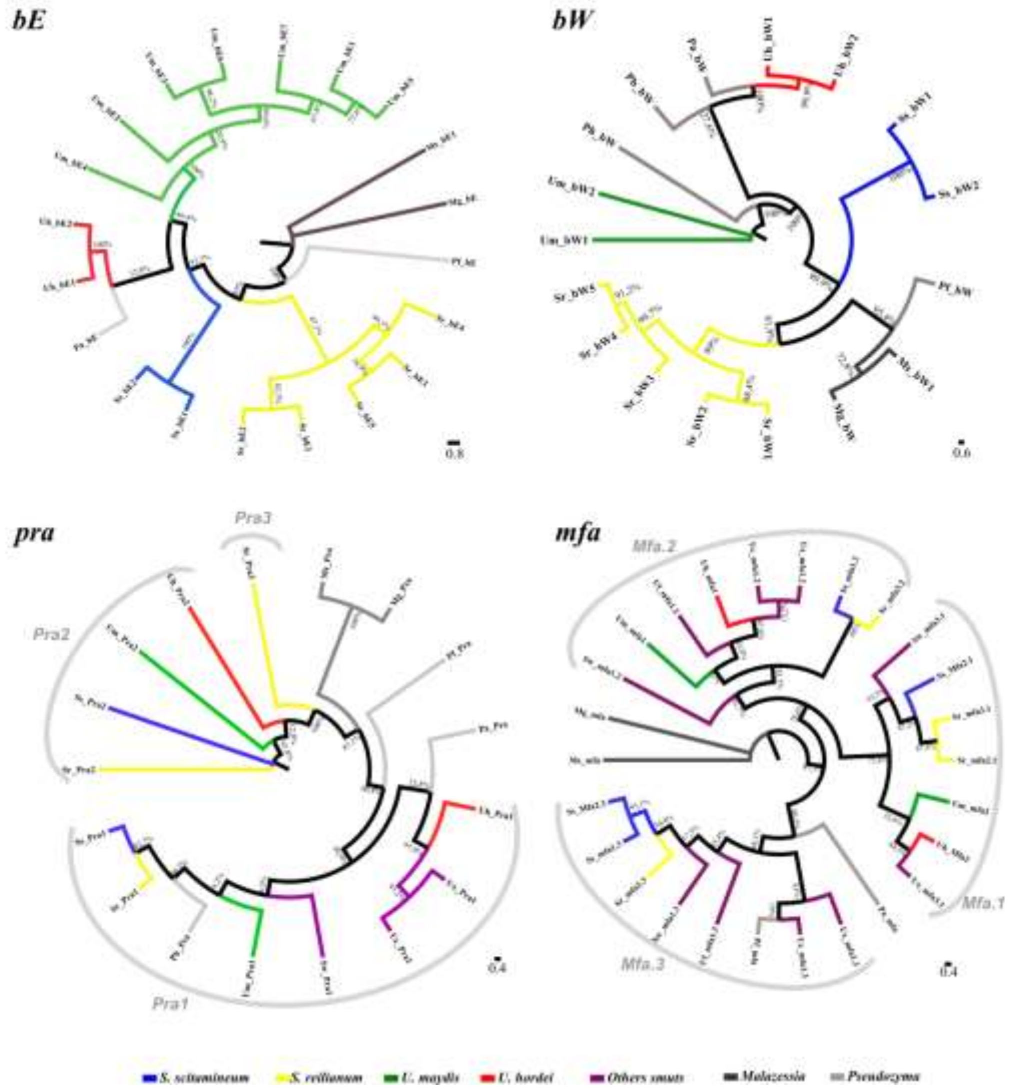

Figure A. Phylogenetic tree for the mating type proteins from related basidiomycetes species obtained by Maximum Likelihood method implemented in PhyML v3.0. T-COFFEE default alignments, amino acid substitution model provided by ProtTest v.3.2, NNI andaLRT SH-like were used in the analysis. The scale bar indicates the number of amino acid substitutions per site. Acession numbers: **bE:** Sr_bE1 (CAI59728.1); Sr_bE2 (CAI59732.1); Sr_bE3(CAI59736.1); Sr_bE4 (CAI59740.1); Sr_bE5 (CAI59744.1); Um_bE1 (EAK81226.1); Um_bE3 (P22017.1); Um_bE4 (P22018.1); Um_bE5 (P22019.1); Um_bE6 (P22016.1); Um_bE7 (P22021.1); Uh_bE1 (CAJ42019.1); Uh_bE2 (CAA79216.1); Pa_bE (GAC73813.1); Pf_bE (XP_007878972.1); Ms_bE1 (AGC24186.1); Ms_bE1 (AGC24186.1); Mg_bE (XP_001731616.1). **bW**: Sr_bW1 (CAI59727.1); Sr_bW2 (CAI59731.1); Sr_bW3 (CAI59735.1); Sr_bW4 (CAI59739.1); Sr_bW5 (CAI59743.1); Um_bW1 (EAK81227.1); Um_bW2 (AAA34221.1); Uh_bW1 (CAJ42018.1); Uh_bW2 (CAA79217.1); Pa_bW (AC73812.1); Pf_bW (XP_007878973.1); Ph_bW (GAC99319.1); Pb_bW (EST06506.1); Ms_bW1 (AGC24185.1); Mg_bW (XP_001731615.1). **Pra**: Sr_Pra1 (CAI59749.1); Sr_Pra2 (CAI59755.1); Sr_Pra3 (CAI59763.1); Um_Pra1 (EAK83421.1); Um_Pra2 (AAA99768.1); Uh_Pra1 (CAJ41875); Uh_Pra2 (AAD56044.1); Sw_Pra1 (AEY62480.1); Ux_Pra1 (AEY62504.1); Uc_Pra1 (AEY62485.1); Pa_Pra (GAC74678.1); Pf_Pra (XP_007877412.1); Pb_Pra (EST05906.1); Ms_Pra (AGC13097.1); Mg_Pra (XP_001731696.1); **Mfa**: Sr_mfa2.1 (CAI59758.1); Sr_mfa2.3 (CAI59748.1); Sr_mfa3.1 (CAI59764.1); Sr_mfa3.2 (CAI59762.1); Um_mfa1 (XP_758529.1); Um_mfa2 (P31963.1); Uh_mfa1 (AAC02682.1); Uh_mfa2 (AF184069.1); Uc_mfa1.2 (AEY62483.1); Uc_mfa1.3 (AEY62484.1); Ux_mfa1.2 (AEY62502.1); Ux_mfa1.3 (AEY62503.1); Ux_mfa3.1 (AEY62543.1); Uf_mfa1.2 (AEY62489.1); Uf_mfa1.3 (AEY62490.1); Sw_mfa1.2 (XP_758529.1); Sw_mfa1.3 (AEY62479.1); Sw_mfa3.1 (AEY62524.1); Pa_mfa; Pf_mfa (XP_007877543.1); Ms_mfa (AGC13096.1); Mg_mfa (XP_001731695.1).*S. scitamineum* proteins were obtained from the genome and from the opposite mating-type strain using a BAC library: Ss_bE1 (g971_chr02_Ss); Ss_bE2 (BAC); Ss_bW1 (g970_chr02_Ss); Ss_bW2 (BAC); Ss_Pra1 (g989_chr2_Ss.1); Ss_Pra2 (BAC); Ss_mfa1.2 (g988_chr02_Ss.1); Ss_mfa1.3 (g988_chr02_Ss.2); Ss_mfa2.1 (BAC), Ss_mfa2.3 (BAC).

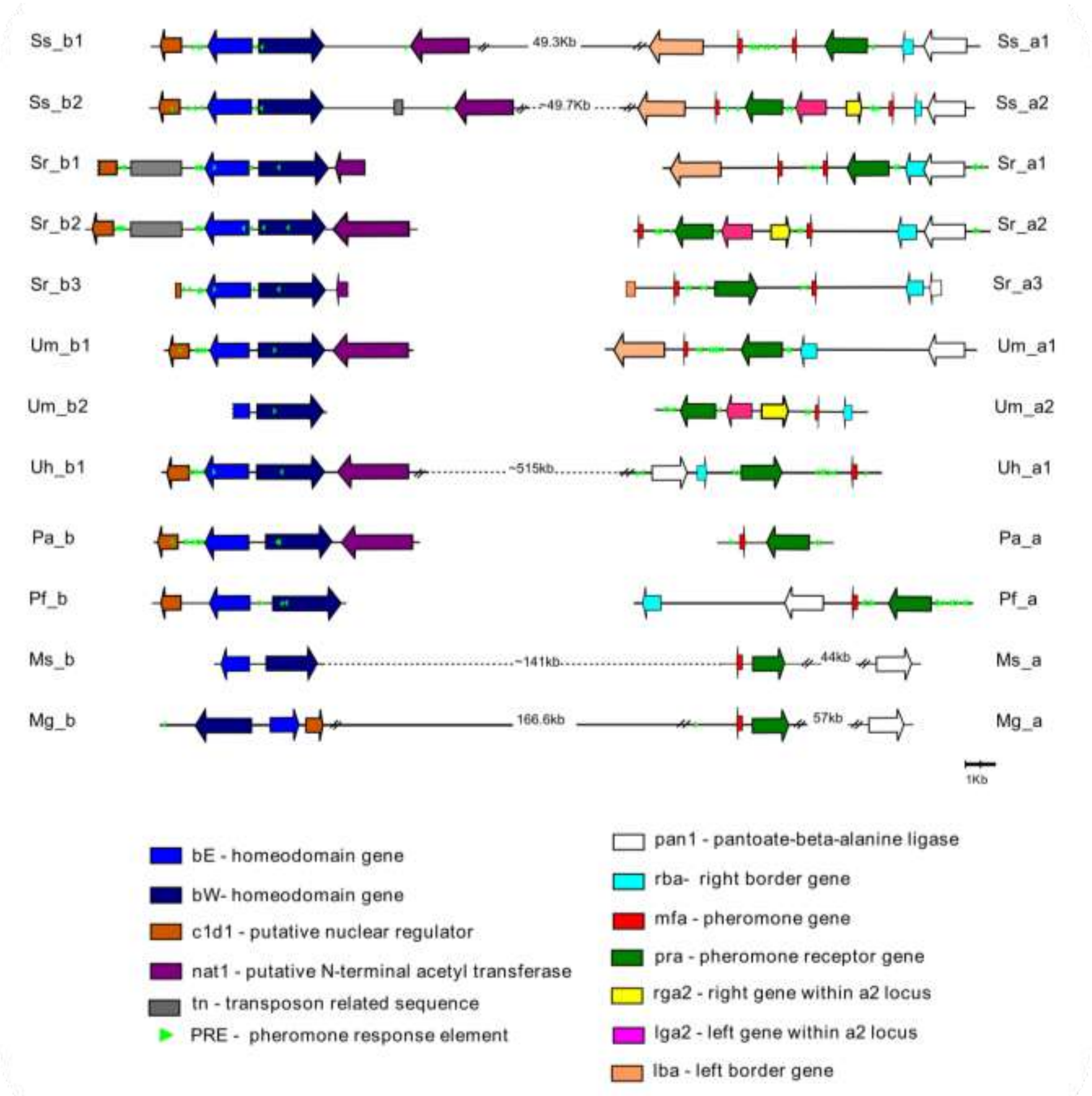

Figure B.Genomic context of mating-type genes from related fungi species. The species can be identified by the initial letters of their scientific nomenclature (*S. scitamineum*, *S. reilianum*, *U. maydis*, *U. hordei*, *Pseudozymaantarctica*, *Pseudozymaflocculosa*,*Malasseziasympodialis*, *Malasseziaglobosa*). Genes are indicated by arrows which orientation shows the guidance of transcription. The same colors indicate probable orthologous genes and their functions are listed at the lower position of the figure. The pheromone response elements (consensus sequence ACAAAGGGA) are also represented in the scheme. The distance between linked loci is indicated and is not drawn to scale. Accession numbers for **b loci** were: Sr_b1 (AJ884583); Sr_b2 (FQ311430.1), Sr_b3 (AJ884585), Um_b1 (AACP01000013), Um_b2 (M84182), Uh_b1 (CAGI01000150.1), Pa_b (BAFG01000292.1), Pf_b (AOUS01000449), Ms_b (CANK01000008), Mg_b (AAYY01000003.1). For **a loci**: Sr_a1 (AJ884588.1), Sr_a2 (FQ311442.1), Sr_a3 (AJ884590), Um_a1 (AACP01000083), Um_a2 (UMU37796), Uh_a1 (CAGI01000150.1), Pa_a (BAFG01000390.1), Pf_a (AOUS01000182), Ms_a (CANK01000022), Mg_a (AAYY01000003.1).
