# Supplementary material for: Complete Genome Sequence of Sporisorium scitamineum and Biotrophic Interaction Transcriptome with Sugarcane
Source: PLoS One. 2015 Jun 12;10(6):e0129318. doi: 10.1371/journal.pone.0129318 (PMC4466345; doi:10.1371/journal.pone.0129318)
Supplement: S7 File — Graphs with GO terms enriched in the S. scitamineum secretome (Figure A). (PDF) [file pone.0129318.s007.pdf]

S7 – Gene Ontology analysis of *S. scitamineum*

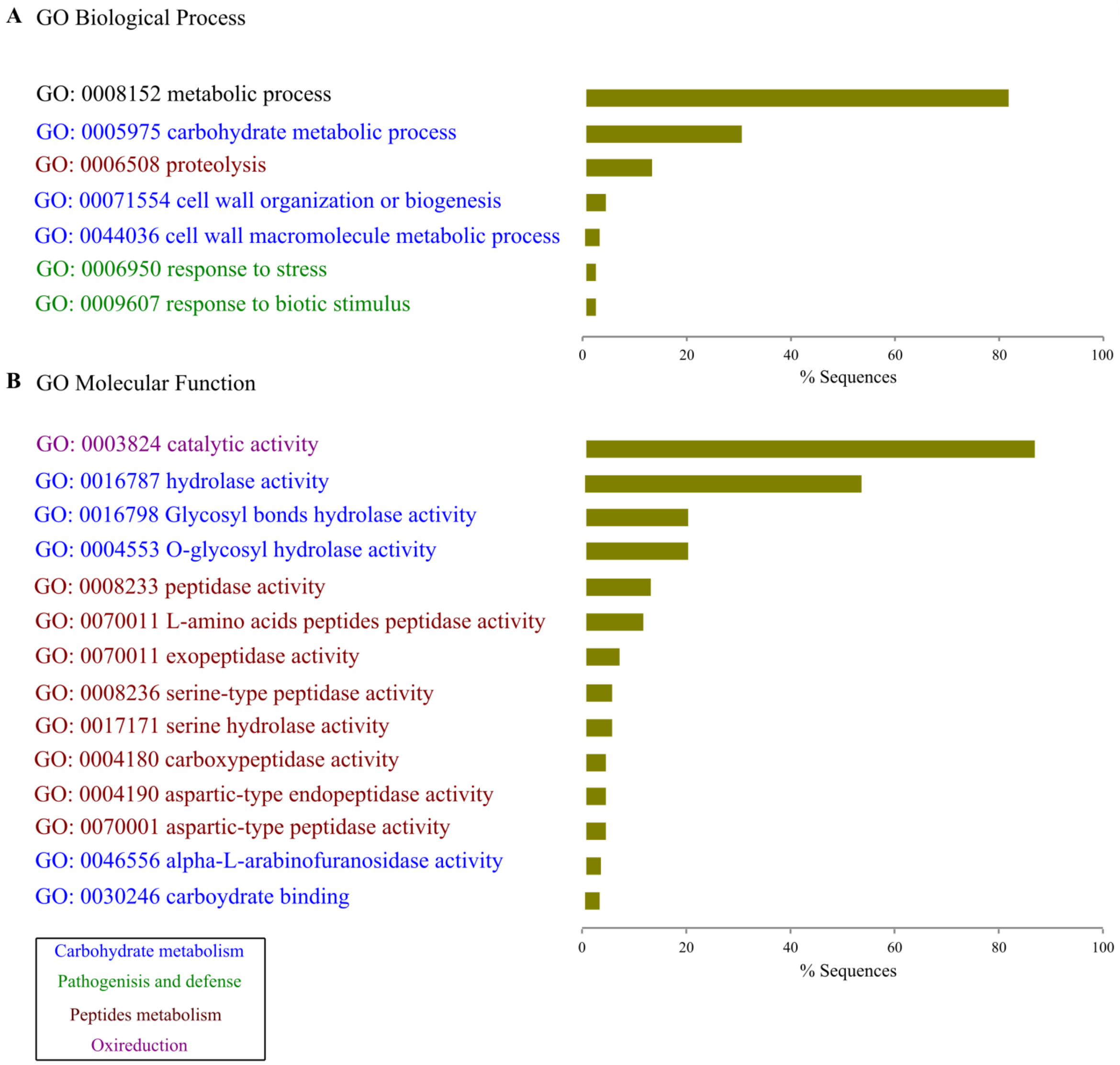

Figure A. Gene Ontologies enriched in the *S. scitamineum* secretome. (A) Biological process and (B) Molecular Function. Graphs represents the percentage of sequences in each GO term enriched in the S. scitamineum secretome (number of proteins with annotation/total number proteins).
